# MultiPrime: A reliable and efficient tool for targeted next‐generation sequencing

**DOI:** 10.1002/imt2.143

**Published:** 2023-10-19

**Authors:** Han Xia, Zhe Zhang, Chen Luo, Kangfei Wei, Xuming Li, Xiyu Mu, Meilin Duan, Chuanlong Zhu, Luyi Jin, Xiaoqing He, Lingjie Tang, Long Hu, Yuanlin Guan, David C. C. Lam, Junbo Yang

**Affiliations:** ^1^ School of Automation Science and Engineering, Faculty of Electronic and Information Engineering Xi'an Jiaotong University Xi'an China; ^2^ MOE Key Lab for Intelligent Networks & Networks Security, Faculty of Electronic and Information Engineering Xi'an Jiaotong University Xi'an China; ^3^ Department of Research and Development Hugobiotech Beijing China; ^4^ Department of Mechanical and Aerospace Engineering The Hong Kong University of Science and Technology Hong Kong China; ^5^ Shenzhen Branch, Guangdong Laboratory of Lingnan Modern Agriculture, Genome Analysis Laboratory of the Ministry of Agriculture and Rural Affairs, Agricultural Genomics Institute at Shenzhen Chinese Academy of Agricultural Sciences Shenzhen China

**Keywords:** degenerate primer design, minimal primer set, multiplex PCR, targeted next‐generation sequencing

## Abstract

We present multiPrime, a novel tool that automatically designs minimal primer sets for targeted next‐generation sequencing, tailored to specific microbiomes or genes. MultiPrime enhances primer coverage by designing primers with mismatch tolerance and ensures both high compatibility and specificity. We evaluated the performance of multiPrime using a data set of 43,016 sequences from eight viruses. Our results demonstrated that multiPrime outperformed conventional tools, and the primer set designed by multiPrime successfully amplified the target amplicons. Furthermore, we expanded the application of multiPrime to 30 types of viruses and validated the work efficacy of multiPrime‐designed primers in 80 clinical specimens. The subsequent sequencing outcomes from these primers indicated a sensitivity of 94% and a specificity of 89%.

## Highlights

- MultiPrime is a user‐friendly and one‐step tool for designing targeted next‐generation sequencing primer sets.
- It integrates degenerate primer design theory with mismatch handling, resulting in improved accuracy and specificity in detecting broad‐spectrum sequences.
- It outperformed conventional programs in terms of run time, primer number, and primer coverage.
- The versatility and potential of multiPrime are highlighted by its potential application in detecting single or multiple genes, exons, antisense strands, RNA, or other specific DNA segments.

## Graphical abstract

This study introduces multiPrime, a novel tool tailored for the wide‐ranging detection of target sequences through targeted next‐generation sequencing (tNGS). By integrating degenerate primer design principles with effective mismatch handling, multiPrime demonstrates enhanced precision and specificity in the identification of diverse sequence types. This breakthrough presents a prospective pathway for optimizing the development of tNGS. In performance comparison, multiPrime excelled over conventional tools in terms of execution time, primer coverage, and the number of candidate primers. It offers a streamlined and versatile solution for the rapid and cost‐effective detection of diverse microbiomes.

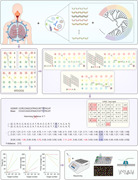

## INTRODUCTION

Targeted next‐generation sequencing (tNGS) has become an increasingly important strategy for exploring the crucial roles of the microbiome due to its high speed, cost efficiency, and broad range. Metagenomic next‐generation sequencing (mNGS) [1, 2, 3] and metatranscriptomic next‐generation sequencing [4, 5] allow a comprehensive analysis of the microbiomes. However, these methods encounter challenges, such as human and environmental microbial genome contamination, necessitating substantial read counts and sample sizes. As a result, these techniques are time‐consuming, expensive, and always challenging to interpret. Though host DNA depletion during DNA extraction has been employed to mitigate human genome contamination [6, 7], their feasibility in resource‐limited and urgent scenarios remains limited, echoing the situation with hybridization‐based next‐generation sequencing. In contrast, polymerase chain reaction (PCR)‐based tNGS [8, 9] is a simple and cost‐effective method to enrich multiple target sequences simultaneously [10]. This method enables rapid amplification of known microbial genomes, as well as virulence or drug resistance genes, from small sample size, yielding results within hours. For example, PCR amplification of rRNA gene sequences using broad‐taxonomic‐range primers has been instrumental in microbial community composition analysis [11, 12, 13, 14]. Yet, the complexity of some microbiomes, such as the virome, presents unique challenges due to the absence of conserved regions and the vast genetic diversity [15, 16]. Consequently, the application of tNGS to such cases requires innovative approaches.

Critical to the success of tNGS is primer design, while factors like melting temperature, GC content, and secondary structure play pivotal roles in the efficient annealing of primers and target [17, 18, 19, 20, 21, 22]. Many software programs have been developed to assist in primer design [23] but few are tailored to the specific needs of tNGS. Existing methods can be categorized into three groups. Conventional PCR primer design tools prove laborious for large‐scale target sets [24, 25, 26], while degenerate primers from sequence alignments [27, 28, 29, 30, 31, 32, 33, 34, 35, 36, 37, 38, 39] or K‐mer generation [40, 41, 42, 43] require a delicate balance between coverage and degeneracy [31, 36, 44]. Machine learning algorithms [45, 46, 47, 48] and other approaches [49, 50, 51] have been explored but often encounter limitations when confronted with the challenge of handling degenerate bases and intricate target sequences. Despite these efforts, the challenge of designing primers for large and diverse targets persists.

To fill this critical gap, we introduce multiPrime, a novel tool developed for multiplex PCR of diverse nucleotide sequences. The main point of its innovation is an integrated mismatch‐tolerant methodology. At its core, multiPrime leverages the fundamental Watson‐Crick hybridization between complementary bases, a process that inherently accommodates minor mismatches. Despite these variations, the prevailing thermodynamic stability of accurately paired bases typically takes precedence over unpaired ones [52]. This mechanism gives rise to primer‐template mismatch annealing—a dynamic that, although it may affect PCR efficiency, does not hinder the amplification process outright. Empirical support from a plethora of studies underscores the robustness of this approach, placing it on par with the outcomes attained through perfect primer‐template annealing [50, 53, 54, 55, 56, 57, 58], such as established amplification refractory mutation system PCR (ARMS‐PCR) [59]. In the dynamic landscape of nucleotide sequence analysis, multiPrime aims to strike a delicate balance between the specificity and efficiency of primers [60]. It further advances the field by seamlessly integrating permissive mismatches into primer design strategies [61, 62], thereby enhancing the dimensions of the primer set—its size, coverage, and efficiency. This synergistic approach opens up promising avenues, offering a nuanced solution that adeptly navigates the complexities posed by expansive and varied target sequences. Within the framework of our study, we have harnessed the power of viruses as a rich and immensely diverse genetic source to establish this innovative methodology. This methodology paves the way for uncomplicated ultra‐multiplex PCR, presenting a potent tool that holds potential not only within the realm of virome but also for the broader application of tNGS across diverse nucleotide sequences. Through this innovative approach, we embark on a journey toward advancing tNGS methodologies, offering a streamlined and versatile solution for the rapid and cost‐effective detection of diverse microbiomes, as well as for the comprehensive unraveling of the intricacies inherent in such diverse microbiotic ecosystems.

## RESULTS

### Target amplification through primer‐template mismatch annealing

To assess the effect of primer‐template mismatches annealing on target amplification, we partitioned primer into three equal nonoverlapping regions: the 5′ end (5′), middle (Mid), and 3′ end (3′). We then designed primers with 0–1 mismatches in these regions to evaluate their ability of target amplification on the Oxford Nanopore Technology (ONT) platform. Our results showed that primers with one mismatch worked effectively (Supporting Information: Figure S5A,B). However, the efficiency of PCR was reduced, with the greatest impact observed when the mismatch was located in the 3′ end (70% and 53%, Supporting Information: Figure S5B). Next, we designed primers with up to two mismatches in the middle and 5′ positions and examined the efficiency of the primers. We found that these primers worked well, with a relative efficiency of at least 68% (Supporting Information: Figure S5B). To further investigate the effects of mismatch number on target amplification, we modified a universal influenza primer, M30F2 [63], by introducing a single nucleotide substitution (A–G) at the 3′ end. This modified primer was named M30F2‐mis. Two primer sets were utilized, each containing only one of the two primers, to evaluate the efficiency of both primers independently. The primers were tested with single‐end 75 sequencing and we observed that primers with 0, 1, and 2 mismatches (F0, F1, and F2 of both primers and F3 of M30F2, Supporting Information: Figure S5D) displayed relatively high efficiency (≥61%), indicating that the primers could function well despite imperfect annealing (F0, F1, and F2 of M30F2‐mis, Supporting Information: Figure S5D). The average relative efficiency of F3 (three mismatches) in M30F2‐mis was 56%, whereas it was only 2% in one of the three replicates (Supporting Information: Table S1). These findings suggest that three mismatches could significantly reduce PCR efficiency. Additionally, our observations demonstrate a comparable performance between primers containing mismatches and those devoid of mismatches. Notably, we also identified a reduction in dimer formation within the primer set (Supporting Information: Figure S6). Overall, our results indicated that one or two mismatches located at the 5′ end or middle regions of primers have a marginal impact on primer‐template annealing.

### MultiPrime significantly reduces the size of primer sets while enhancing the coverage and compatibility of these sets

**Figure 1:**
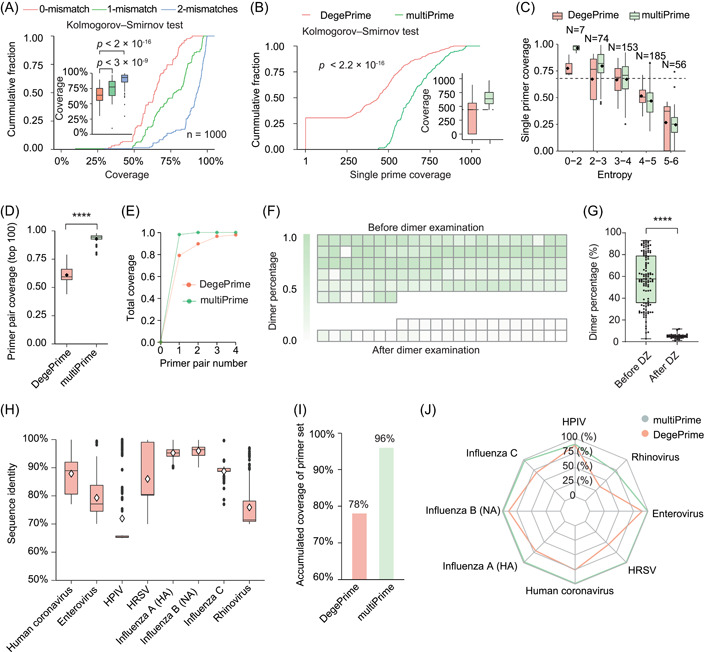
MultiPrime efficiently broadens the spectrum and enhances compatibility. (A) The coverage of primers designed by multiPrime using 1000 sequences, allowing for 0, 1, and 2 mismatches. (B) The cumulative fraction of single primer coverage for primers designed by multiPrime and DegePrime. (C) The coverage of single primers designed by multiPrime and DegePrime in different entropy regions. (D) The top 100 primer pair coverage values for DegePrime and multiPrime. (E) The number of primer pairs required to achieve satisfactory coverage. A heatmap (F) and boxplot (G) were used to show that the percentage of dimers was significantly reduced by dimer examination. Before dimer examination (Before DZ) was defined as the primer set combination without dimer examination. After dimer examination (after DZ) was defined as the primer set combination with dimer examination. (H) The sequence identity of the eight viruses was used for validation. (I) The accumulated coverage of the core primer set was evaluated across all eight viruses. The primer set was designed by multiPrime (v2.0.2) with the following parameters: identity: 0.8; seq_number_ANI: 60; drop: “T”; coordinate: 0; Others: Default. For detailed definitions of the parameters, see YAML files on GitHub. (J) Accumulated coverage of each individual virus by the primer set. HPIV, human parainfluenza virus; HRSV, human respiratory syncytial virus. ****p < 0.0001 by *t*‐test (two‐sided).

MultiPrime designs primers that can tolerate up to X (0, 1, or 2) mismatches, thereby reducing degeneracy and improving coverage. For example, to cover all 10 input sequences (Supporting Information: Figure S7A), a primer with a high dependency value of 512 would be required (Supporting Information: Figure S7B). Alternatively, by allowing for a single mismatch in primer design, four potential primers were identified that could cover all 10 targets with a significantly lower degeneracy of 4. To investigate the extent of achievable improvement through mismatch tolerance, we designed primers for 1000 sequences of human rotavirus A by allowing for 0, 1, and 2 mismatches (Figure 1A). In silico results showed that primers with 1 or 2 mismatches significantly improved primer coverage, demonstrating that using fewer primers with mismatch tolerance can lead to a broader spectrum of coverage. In terms of the ability to design degenerate primers, we compared the performance of multiPrime's primer design module with that of DegePrime—a widely used program for designing degenerate primers for bacterial species that has been shown to outperform other programs such as HYDEN. We discovered that the primers designed by multiPrime with one mismatch tolerance had significantly higher coverage than those produced by DegePrime (Figure 1B), particularly when the entropy of the primer region was less than 3 (Figure 1C). This advantage was even more pronounced when comparing combinations of primer pairs (Figure 1D). The results showed that the multiPrime primer design module yielded higher‐coverage primers with either one primer pair (98.7%) or two primer pairs (100%), while DegePrime required at least four primers to attain comparable coverage (97.8%). Additionally, we embarked on an iterative primer design using DegePrime for the uncovered sequences. Regrettably, this approach was impeded by primer compatibility concerns, thwarting the integration of supplementary primers to augment coverage (Figure 1E).

While reducing the number of primers in primer sets using multiPrime can significantly decrease the likelihood of primer incompatibility, dimer formation remains a significant challenge. To address this challenge, we employed an empirical loss function to assess potential dimers in the primer set. We optimized the threshold of the loss function using 154 next‐generation libraries from five batches with an average 213‐plex PCR system designed by multiPrime (Supporting Information: Figure S8). Our results indicated that the proportion of dimers was significantly reduced from 57.23 ± 23.5% (*n* = 117) to 4.97 ± 2.07% (*n* = 37) (Figure 1F,G, Supporting Information: Table S2).

### MultiPrime outperforms conventional methods in primer set optimization for large and diverse sequences

Multiple sequence alignment (MSA) can be inefficient when dealing with large‐scale sequences due to the required computational power and time. Therefore, we investigated the feasibility of randomly selecting a certain number of sequences from each class for primer design. To determine the number of sequences needed to achieve satisfactory coverage, 20,723 CDSs of influenza A were used. We randomly selected 10–2500 input sequences for evaluation using multiPrime to assess the resulting coverage. Our analysis demonstrated that a minimum of 200 input sequences are required to ensure precise primer coverage. Additionally, the number of candidate primers decreased rapidly when the number of input sequences exceeded 1000 (Supporting Information: Figure S9A). Furthermore, the run time for the MSA increased significantly as the number of input sequences increased (Supporting Information: Figure S9B). These findings suggest that the optimal range for efficient and effective primer design is from 200 to 1000 input sequences.

There is no MSA‐dependent software available that can automatically and efficiently design a minimal primer set for the 43,016 sequences derived from eight different viruses currently. To address this limitation, we modified multiPrime by replacing its primer design module with DegePrime. We then conducted a comparative analysis of the performance of the modified multiPrime and the original version. The high diversity of the input sequences was demonstrated by the identity of each virus (Figure 1H). Our analysis revealed that multiPrime's primer design module outperformed DegePrime in terms of primer design efficiency. In particular, multiPrime's primer design module required only 17 min to design primers, while DegePrime required 48 min. The primer set designed by multiPrime achieved a coverage of over 96% and 95%, with different parameters, significantly higher than that achieved by the DegePrime modified version (78% and 69%, respectively) (Figure 1I, Supporting Information: Table S3). The coverage achieved by multiPrime's primer set for each virus was also significantly higher than that of the DegePrime primer set (Figure 1J). We also compared our tool with Primux, which is a K‐mer creation‐dependent tool. However, Primux encountered a segmentation fault and crashed after running for approximately 70 h. These results indicate that multiPrime is effective in dealing with large and diverse sequences, while conventional primer design software such as DegePrime or Primux is inadequate, even when combined with our strategy.

### Experimentally validating primer‐target amplicons using a long‐read sequencing platform

**Figure 2:**
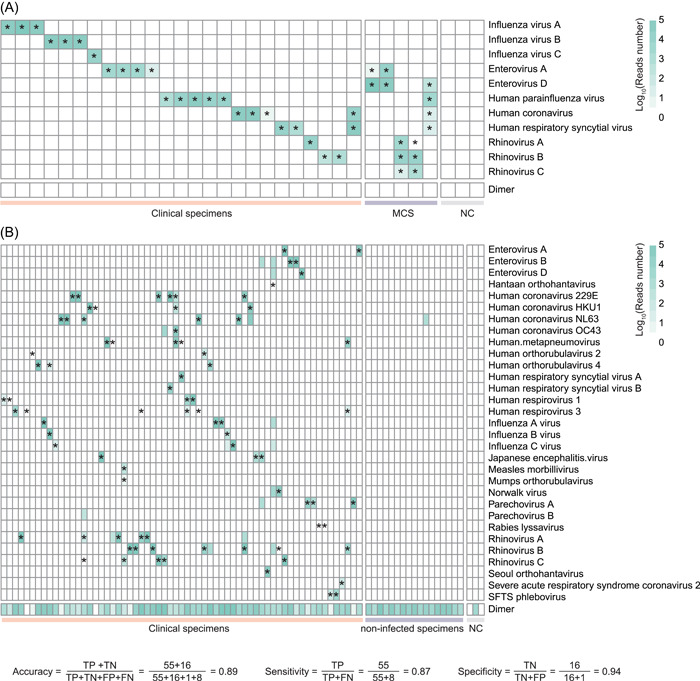
Validation of two primer sets designed by multiPrime using two sequencing platforms. (A) Validation of the primer set on the ONT platform. MCS, mixed clinical specimens (2–4 clinical specimens in one tube); NC, negative control; stars indicate the target virus. (B) Validation of the primer set on the NextSeq. 500 platform. The primer set was designed by multiPrime (v2.0.3) with the following parameters: coordinate: 4; seq_number_ANI: 10; Others: Default. Clinical NC, normal clinical specimen, which refers to samples obtained from healthy individuals devoid of any viral sequences; NC, negative control refers to intentionally empty samples without any template; stars indicate the target virus.

To verify whether the target sequences were being amplified as designed, we utilized the primer set to amplify PCR products from 30 clinical specimens and three negative control samples and subsequently sequenced the product using a long‐read sequencing platform (Oxford Nanopore Technology [ONT]). All viruses were accurately identified with the target amplicon, and no virus reads were detected in the negative controls, indicating the high specificity of the primer set (Figure 2A, Supporting Information: Table S4). Furthermore, the detection of two target viruses in one clinical specimen and 2–4 clinical specimens in one tube demonstrated the capability of the primer set to detect complex infections, which is crucial for accurate diagnosis and treatment.

We then assigned all target reads to their primers and estimated the effectiveness of the primers by comparing them to primers that were perfectly annealed. Our analysis revealed that the number of mismatches between the primer and template DNA plays a vital role in primer‐template annealing efficiency. Moreover, mismatches located at the 3′ end of the primer and 5′‐adjacent positions may lead to a relatively high decrease in primer efficiency (Supporting Information: Figure S10A). To verify the impact of mismatch number and position on primer design, we synthesized a DNA fragment (NC_001488.1:4925‐5423) and constructed a template vector. We designed a degenerate primer and estimated its relative efficiency (Supporting Information: Table S5). Our analysis showed that mismatches located at the 3′ end (–1) had the most significant impact on primer efficiency, while those adjacent to the 5′ end (–17) had a smaller impact than those at the 3′ end. Mismatches in the middle (−12) had the smallest effect (Supporting Information: Figure S10B).

### Experimental validation of the primer set's efficacy in capturing diverse sequences

**Figure 3:**
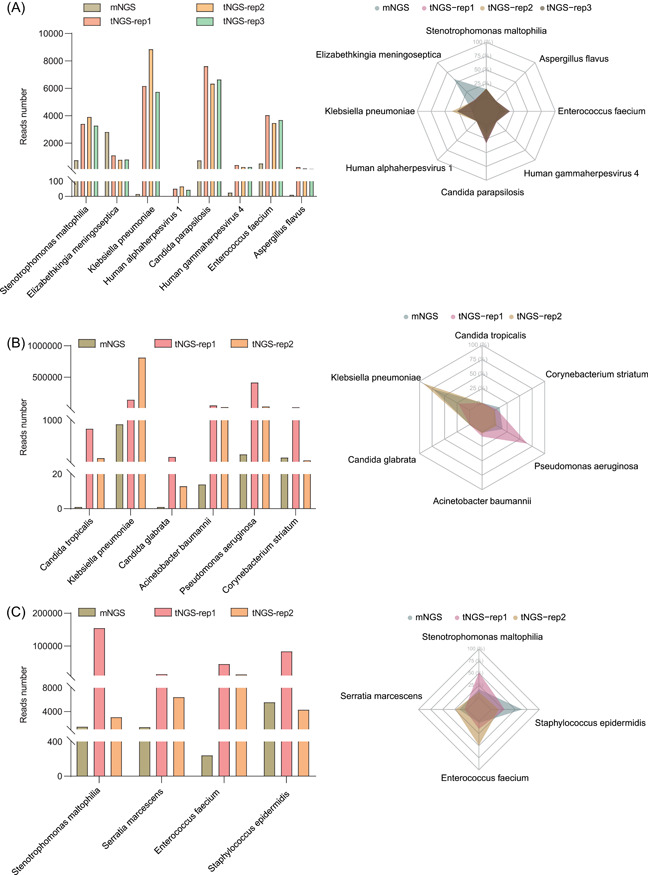
The efficacy of the primer set in capturing diversity sequences. Target read number (barplot, left) and percentages (radarplot, right) were obtained from both metagenomic next‐generation sequencing and targeted next‐generation sequencing analyses for three clinical specimens: clinical specimen 1 (A), clinical specimen 2 (B), and clinical specimen 3 (C). These specimens collectively contained a total of 16 pathogens, including fungi and bacteria.

To assess the efficacy of the primer set in capturing diversity and abundance, we devised primers targeting 16 epidemic pathogens and contrasted the outcomes with those of mNGS using three samples. The results demonstrated that our primer set facilitated target detection at a level comparable to mNGS. Notably, all designated targets were successfully identified, with their corresponding read counts significantly surpassing those obtained through mNGS (Figure 3A–C, Supporting Information: Table S6). These findings underscore the effectiveness of the multiPrime‐designed primer set in capturing both the diversity and abundance of targeted sequences.

### Application of multiprime in the development of a primer set for tNGS for 30 viruses

To further confirm the accuracy and effectiveness of the primer set designed by multiPrime, we designed a larger set of primers targeting 30 viruses, ensuring that 3′ end mismatches were avoided. Subsequently, we evaluated this larger primer set using a cohort of 80 clinical specimens. Our results showed that the primer set was able to detect viruses using next‐generation sequencing with a single‐end 75 sequencing model (Figure 2B, Supporting Information: Table S7). The primer set yielded a sensitivity of 87%, a specificity of 94%, and an accuracy of 89% (Supporting Information: Table S8). Among the 63 infected specimens, only *Hantaan orthohantavirus* and *rabies lyssavirus* remained unidentified, while all other viruses were correctly identified with an accuracy score exceeding 95% (Supporting Information: Table S9). Subsequently, alternative cluster‐specific primers were selected for these two viruses. The primer set was subjected to re‐evaluation. Remarkably, both of these viruses were successfully identified during this subsequent assessment (Supporting Information: Table S7). These results demonstrated that multiPrime‐designed primers are reliable and precise in detecting viruses. In addition, we also assigned virus reads to their corresponding primers to evaluate the efficiency of each primer. Our analysis revealed that the number of mismatches remains the most significant factor affecting primer efficiency. Specifically, we found that positions adjacent to the 5′ end (while avoiding the 3′ end) had a relatively high impact compared to other positions (Supporting Information: Figure S10C).

## DISCUSSION

The effectiveness and detection capabilities of tNGS rely heavily on the primer set used, making the enhancement of primer coverage a major focal point. The naive approach for tNGS primer sets candidate (PSC) would be the “divide and conquer” procedure. This approach involves dividing the input sequences into two groups, one containing primer and the other without, and repeating the procedure for the nonprimer‐containing group until all sequences are matched or no candidate primers can be found. However, it is challenging and time‐consuming to design primers and assess their compatibility at each iteration. It is crucial to ensure that newly designed primers do not interact with existing ones to prevent off‐target amplification and dimer formation. To address these challenges, an effective approach would be to group input sequences based on their identity, design primers for each group, and combine intergroup primers into a primer set. However, the success of this approach depends on achieving sufficiently high primer coverage for each group. If the primer coverage is insufficient or unsatisfactory, this approach would not be feasible. MultiPrime aims to design a degenerate primer that is similar to all input sequences and matches well with them by tolerating up to two mismatches instead of requiring a perfect matching. Compared to conventional primer design programs, multiPrime yields a higher number of candidate primers with higher coverage, resulting in a more efficient primer set with fewer primers. Furthermore, multiPrime uses a loss function to evaluate the likelihood of dimer formation, resulting in a reduced probability of dimer formation within the primer set and facilitating expansion of the primer set as needed. Overall, multiPrime offers an innovative and efficient approach for one‐step primer set design for tNGS.

PCR failure can be attributed to various factors, such as contamination level, poor template quality, inadequate operator proficiency, or suboptimal PCR conditions. Therefore, it is essential to validate all primer pairs in the primer set and monitor their amplification efficiency to ensure accuracy. Moreover, altering reagents or the reaction system for a specific primer pair may influence the amplification of other primers, thus requiring additional primer pair validation. MultiPrime offers a cluster‐specific set of candidate primer pairs that can complement or substitute existing primer pairs in the primer set, empowering researchers to design more effective panels to meet their research and development demands.

## CONCLUSIONS

In our study, we have demonstrated that primers with specific mismatches can accurately amplify targets. MultiPrime incorporates a mismatch‐tolerant feature that simplifies the process of managing mismatches. It can design primers while avoiding mismatches at specific positions and automatically generate two minimal primer sets for tNGS. In a small cohort, direct sequencing using ONT identified all viruses while not detecting any nontarget virus reads in negative controls, proving the ability of primers with mismatch tolerance to identify target viruses. In a larger cohort, we achieved high identification accuracy, sensitivity, and specificity. Overall, multiPrime significantly expands the range of target sequences and enables ultra‐multiplex PCR. Incorporating multiPrime into tNGS is expected to simplify and enhance the effectiveness of the technique.

## MATERIALS AND METHODS

### Implementation

**Figure 4:**
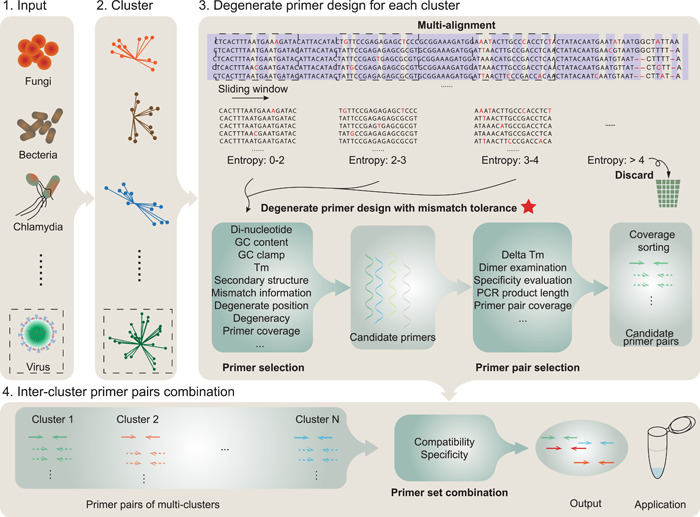
Schematic diagram depicting the one‐step targeted next‐generation sequencing (tNGS) primer set design method developed by using multiPrime. The red star indicates the step where the method is described in more detail. MultiPrime comprises four primary stages: (1) Input: In this initial phase, the collection of target sequences is required. (2) Cluster: Redundant sequences are eliminated and clusters are established based on sequence identity. (3) Degenerate Primer Design for Each Cluster: Utilizing MUSCLE or MAFFT, a multialignment procedure is conducted, followed by the design of candidate primers using the nearest‐neighbor model. (4) Intercluster Primer Pair Combination: Primer pairs are selected considering factors such as PCR product length, melting temperature, dimer formation assessment, coverage despite errors, and other pertinent criteria. Subsequently, a greedy algorithm is employed to merge primer pairs into an optimal minimal primer set, guided by dimer formation analysis.

The workflow of multiPrime is illustrated in Figure 4 and the bioinformatic pipeline is illustrated in Supporting Information: Figure S1. The manual and software are freely accessible to all users at https://github.com/joybio/multiPrime. The installation video for multiPrime is available on Figshare (https://figshare.com/articles/media/Installation_video_of_multiPrime/23904159). All scripts in multiPrime are implemented as Python scripts, and the joint pipeline is written in Snakemake [64]. The input of multiPrime is a FASTA format file. It can be a set of CDSs, genes, genomes, or other kinds of sequences. MultiPrime has three main steps: First, multiPrime classifies the sequences by identity [65, 66]. Second, multiPrime randomly selects N (default: 500) sequences from each class to generate multi‐alignment results [67, 68] and design degenerate primers for them by allowing mismatches through the nearest‐neighbor (NN) model. Finally, a greedy algorithm is used to combine intercluster primer pairs into one primer set according to dimer examination and specificity evaluation.

#### IUPAC transition table

We implemented a hash table to simplify the NN model (Supporting Information: Figure S2A). In this table, degenerate base values are derived from normal bases, and the features of the IUPAC transition (trans) table are as follows:

The value of each normal base [A, G, C, T] is unique.

The value of a degenerate base is the sum of the contained normal bases.

The value of each degenerate base is unique.

The rounded down value of each base is equal to its degeneracy.

The value of a degenerate base minus the value of a contained normal base is present in the IUPAC trans table but it will not be when the value of a noncontained normal base is subtracted.

#### Nearest‐neighbor model

The nearest‐neighbor (NN) model's goal is to identify a degenerate primer that can match as many sequences as possible with up to X (0, 1, or 2) mismatches. It takes the multialignment result as input and identifies primer‐length windows as candidate primers. When a window begins or ends with a “‐” character, “‐” is removed, and the window shifts to the next base. The window is then extended to match the length of the primer being sought. However, not all windows are suitable for degenerate primer design (DPD). To filter out high‐entropy windows unlikely to yield effective primers, we employ entropy as a metric to quantify variation within the window. Only those windows with entropy values below 3.6 are selected for further processing in the DPD protocol (Supporting Information: Figure S2B).

Typically, the most frequent sequence or the most frequent bases in each position will be used as the primary primer (which can be called the optimal primer) to initialize DPD. However, the NN model assumes that both the base frequency at each position and the weight between NN bases contribute to optimal primer selection, and the Viterbi algorithm [69] (Supporting Information: Figure S2C) is employed to find the optimal primer. In certain instances, the most frequently occurring sequence may differ substantially from the most probable one. Therefore, we utilize both the most frequent sequence and the Viterbi result as the optimal primer to initiate DPD to select the best primer. A simplified version of our algorithm is outlined as follows: First, we compute the frequency and NN arrays of the input sequences (Supporting Information: Figure S3A,B). Second, we initialize DPD using the NN model until acceptable coverage is achieved with errors or degeneracy of the primer not exceeding the threshold (Supporting Information: Figure S3C,D). Third, we identify candidate primer pairs based on criteria, such as coverage with errors, evaluation of dimers, Tm difference between primers, and additional information. Finally, we obtain eligible candidate cluster‐specific primer pairs.

#### The efficiency of PCR with specific primer

We assigned all target reads to their primers and estimated the effectiveness of the primers by comparing them to primers that were perfectly annealed.

$$\text{Efficiency}\;(\text{primer}-i)=\frac{\text{Reads number}\;(i)}{\text{Reads number}\;(c)}.$$

Efficiency (primer‐i) refers to the PCR efficiency specifically associated with primer‐i. Reads number (i) represents the count of reads that correspond to the target region of primer‐i. Similarly, reads number (c) denotes the quantity of reads attributed to the target region of the primer with a perfect match.

#### Evaluation of degenerate primer coverage with errors

We introduce a new metric, the Y‐distance, which is an extension of the Hamming distance (Supporting Information: Figure S4A), to measure the distance between degenerate primer and target sequence based on the number and positions of noncontained bases. For example, “G2SKR” is a common primer for Norovirus detection [70, 71, 72], and “Base” is a hypothetical target sequence. The Hamming distance between “G2SKR” and “Base” is 6, which is not sufficient for describing the difference because there is only a one‐nucleotide difference between “Base” and the closest sequence contained in “G2SKR.” However, there is only one value in the Y‐distance that is not in the IUPAC trans table (Supporting Information: Figure S4B,C), and 17 indicates that the 17th position in “G2SKR” is different than the 17th position in “Base.” The thresholds of the Y‐distance in multiPrime are as follows:

1) The Y‐distance length, indicating the number of allowable mismatches, should be limited to fewer than 2.
2) Certain positions (such as the 3′ end of the primer) should be avoided in the Y‐distance.

#### Dimer examination

The loss function used in multiPrime is an empirical formula that is modified from badness and is derived from SADDLE [73]. It aims to evaluate the compatibility between two primers. Primers are incompatible if the loss function between any two primers is greater than the threshold (high risk ≥ 3.96 and low risk ≤ 3).

$${\text{Loss function}\;(\text{primer}-\text{primer})=\log}_{10}\frac{2^{\text{length}}*2^{\text{GC content}}}{\left(2^{\text{distance}1}-0.9\right)*\left(2^{\text{distance}2}-0.9\right)}.$$

Length is the length of the complementary sequence, distance1 and distance2 are the distances of the complementary sequence to the 3′ ends of primer, and GC content is the number of G/C nucleotides in the complementary sequence.

The threshold is determined by identifying the inflection point of the dimer ratio (Supporting Information: Figure S8) to balance the specificity and sensitivity of primer pairs, ensuring that the selected primer pairs have minimal dimer formation while achieving high coverage.

$$\text{Dimer ratio}\;(L)=\frac{\text{DNL}}{\text{DNT}},$$

where, *L* is the value of the loss function, DNL is the dimer reads of primer‐primer with a loss function greater than *L*, and DNT is the total dimer reads.

#### Specificity evaluation

The burrows‐wheeler transform (BWT) algorithm [74] is employed to evaluate the specificity of the primer pairs. Primer pairs with 1 mismatch (at least 4 bp away from the 3′ end of the primer) having target regions in the host genome or transcriptome are considered potential nonspecific primer pairs. We use 9 bp of the 3′ terminus for mapping, which means that the 9 bp terminus of primer pairs with a target amplicon in the host should be considered 1 unit off‐target [21].

### Experimental validation

#### Input of multiprime

In the context of third‐generation sequencing, we employed a comprehensive data set consisting of a total of eight pandemic respiratory system viruses along with a combined count of 43,016 complete genomic and CDS (coding sequence) sequences. This data set was obtained from the National Center for Biotechnology Information (NCBI) and featured a diverse array of viral types, including 2522 enteroviruses (A and D, complete genome), 429 human coronaviruses (complete genome), 1155 human respiratory syncytial viruses (complete genome), 27,727 influenza A viruses (CDS), 9129 influenza B viruses (CDS), 166 influenza C viruses (CDS), 509 human parainfluenza viruses (HPIV; complete genome), and 1379 rhinoviruses (A, B, and C, complete genome), and were used as the input of multiPrime. All of these FASTA files were consolidated into a single file, which served as the input for the multiPrime. For the purposes of diversity and abundance analysis, a selection of 16 pandemic pathogens was chosen as the input for multiPrime. Furthermore, as part of our analysis using next‐generation sequencing, we extended our data set to include genomic and CDS sequences from a total of 30 distinct RNA viruses, encompassing various subtypes. This expanded data set was subsequently utilized as input for multiPrime.

#### Output of multiprime

The resulting output comprises a directory encompassing seven subdirectories along with over 40 individual files. A comprehensive elucidation of each file's particulars is available in the provided URL: https://github.com/joybio/multiPrime/README.md. Notably, the primary output encompasses two ultimate primer sets and the cluster‐specific primers.

#### Sample selection and preparation

Clinical specimens (sputum/bronchoalveolar lavage fluid [BALF]/cerebrospinal fluid [CSF]), virus stocks, and negative controls were used to evaluate multiPrime.

For third‐generation sequencing, a total of 19 clinical specimens (four sputum, 14 BALF, and one CSF), five virus stocks (three from Guangzhou BDS Biological Technology Co., Ltd. and two from the China Center for Type Culture Collection), and three negative controls were used for targeted ONT direct sequencing. The target virus sequences of clinical specimens (two influenza B viruses and one influenza C virus) were previously determined by mNGS.

For next‐generation sequencing (SE75), three influenza A BALF samples were used to assess mismatch effects; 63 clinical specimens with a positive RNA virus infection diagnosis with mNGS, 17 normal controls, and three negative controls were used to evaluate the performance of the 94‐plex multiPrime primer set.

RNA was extracted using a Quick DNA/RNA Miniprep Kit (200 Preps) (ZYMO) following the manufacturer's instructions, and the sample was eluted in RNase‐free water.

#### Library and sequencing

Amplicons for next‐generation sequencing were generated using AccurSTART U+ One Step RT‐qPCR Super PreMix (ONE TUBE) (Vazyme) and then purified using AMPure XP (Beckman). The purified amplicons were then amplified using KAPA HiFi HotStart Uracil+ ReadyMix (2X) (Roche) to complete library construction. A modified protocol for the NextSeq. 500/550 High Output Kit v2.5 was used for amplicon sequencing, and sequencing was performed on the Illumina NextSeq. 500 platform (75 cycles). Amplicons for third‐generation sequencing were generated by AccurSTART U+ One Step RT‐qPCR Super PreMix (Vazyme) and then purified using AMPure XP (Beckman). Second‐round PCR was performed with KOD‐Multi & Epi (TOYOBO) to complete library construction, and the library was sequenced on GridION X5.

#### Analysis of next‐generation sequencing data

The RCP pipeline (see Acknowledgments) is utilized for the analysis of next‐generation sequencing data. Briefly, sequencing reads were trimmed by BBMap (https://sourceforge.net/projects/bbmap). Seqkit [75] was employed to extract unique reads, and an R script was used to determine the duplication level. Unique reads were annotated with a simplified NT database (only viruses) by using BLAST (2.13.0+) [76, 77]. Dimer detection and counting were also performed using BLAST (2.13.0+).

#### Analysis of third‐generation sequencing data

Base calling of the raw fast5 files produced by sequencing was performed using Guppy v1.1. Adapters were trimmed off by Porechop (https://github.com/rrwick/Porechop). Clean reads were mapped to the *Homo sapiens* genome assembly T2T‐CHM13v2.0 and YH to remove the host genome with minimap2 [78]. The remaining reads were annotated with a simplified NT database (only viruses) by using BLAST (2.13.0+). “Confident on targets”: Reads annotated to viruses (BLAST). “High confidence on target”: “Confident on targets” reads with specific 5′/3′ primers.

## AUTHOR CONTRIBUTIONS

The project was conceptualized and designed by Junbo Yang. All scripts were written by Junbo Yang. Experiments were conducted by Han Xia, Chen Luo, Junbo Yang, and Yuanlin Guan. The sequencing data were analyzed by Junbo Yang, Kangfei Wei, and Meilin Duan. Junbo Yang, Zhe Zhang, and David C. C. Lam wrote the manuscript. All authors contributed to the article and attested that they meet the current ICMJE criteria for authorship.

## CONFLICT OF INTEREST STATEMENT

The authors declare no conflict of interest.

## ETHICS STATEMENT

Our research was conducted in accordance with the principles of the Declaration of Helsinki. The study was approved by the Human and Artefacts Research Ethics Committee (HAREC) at The Hong Kong University of Science and Technology under the approval number HREP‐2023‐0221. Written informed consent was obtained from all participants before collecting clinical specimens for research purposes. All specimens were stored securely and handled using appropriate procedures to maintain their integrity. Deidentified data were used in all analyses to protect patient privacy.

## Supporting information

Supporting information.

Supporting information.

## Data Availability

All the necessary Python scripts and test data required for replicating our results can be found at https://github.com/joybio/multiPrime. The majority of the raw sequencing data presented in this paper have been deposited in the Genome Sequence Archive (Accession No. CRA009723) at the National Genomics Data Center, China National Center for Bioinformation/Beijing Institute of Genomics, Chinese Academy of Sciences, and are publicly available at https://ngdc.cncb.ac.cn/gsa. However, some data will not be made available in a public repository due to confidentiality concerns related to patient privacy. For further inquiries, please contact the corresponding author, Junbo Yang.

## References

[1] Chen, Xiancheng , Ke Cao , Yu Wei , Yajun Qian , Jing Liang , Danjiang Dong , Jian Tang , Zhanghua Zhu , Qin Gu , and Wenkui Yu . 2020. “Metagenomic Next‐Generation Sequencing in the Diagnosis of Severe Pneumonias Caused by Chlamydia Psittaci.” Infection 48: 535–542. 10.1007/s15010-020-01429-0 32314307 PMC7223968

[2] Gu, Wei , Steve Miller , and Charles Y. Chiu . 2019. “Clinical Metagenomic Next‐Generation Sequencing for Pathogen Detection.” Annual Review of Pathology: Mechanisms of Disease 14: 319–338. 10.1146/annurev-pathmechdis-012418-012751 PMC6345613 30355154

[3] Wooley, John C. , and Yuzhen Ye . 2010. “Metagenomics: Facts and Artifacts, and Computational Challenges.” Journal of Computer Science and Technology 25: 71–81. 10.1007/s11390-010-9306-4 PMC2905821 20648230

[4] Wu, Jieying , Weimin Gao , Weiwen Zhang , and Deirdre R. Meldrum . 2011. “Optimization of Whole‐Transcriptome Amplification From Low Cell Density Deep‐Sea Microbial Samples for Metatranscriptomic Analysis.” Journal of Microbiological Methods 84: 88–93. 10.1016/j.mimet.2010.10.018 21044647

[5] Zhao, Na , Jiabao Cao , Jiayue Xu , Beibei Liu , Bin Liu , Dingqiang Chen , Binbin Xia , et al. 2021. “Targeting RNA With Next‐ and Third‐Generation Sequencing Improves Pathogen Identification in Clinical Samples.” Advanced Science 8: e2102593. 10.1002/advs.202102593 34687159 PMC8655164

[6] Hansen, Wendy L. J. , Cathrien A. Bruggeman , and Petra F. G. Wolffs . 2013. “Pre‐Analytical Sample Treatment and DNA Extraction Protocols for the Detection of Bacterial Pathogens from Whole Blood.” Methods in Molecular Biology 943: 81–90. 10.1007/978-1-60327-353-4_4 23104282

[7] Marotz, Clarisse A. , Jon G. Sanders , Cristal Zuniga , Livia S. Zaramela , Rob Knight , and Karsten Zengler . 2018. “Improving Saliva Shotgun Metagenomics by Chemical Host DNA Depletion.” Microbiome 6: 42. 10.1186/s40168-018-0426-3 29482639 PMC5827986

[8] ElSharawy, Abdou , Jason Warner , Jeff Olson , Michael Forster , Markus B. Schilhabel , Darren R. Link , Stefan Rose‐John , et al. 2012. “Accurate Variant Detection Across Non‐Amplified and Whole Genome Amplified DNA Using Targeted Next Generation Sequencing.” BMC Genomics 13: 500. 10.1186/1471-2164-13-500 22994565 PMC3534403

[9] Li, Shiying , Jin Tong , Yi Liu , Wei Shen , and Peng Hu . 2022. “Targeted Next Generation Sequencing is Comparable with Metagenomic Next Generation Sequencing in Adults with Pneumonia for Pathogenic Microorganism Detection.” Journal of Infection 85: e127–e129. 10.1016/j.jinf.2022.08.022 36031154

[10] Gaston, David C , Heather B. Miller , John A. Fissel , Emily Jacobs , Ethan Gough , Jiajun Wu , Eili Y. Klein , Karen C. Carroll , and Patricia J. Simner . 2022. “Evaluation of Metagenomic and Targeted Next‐Generation Sequencing Workflows for Detection of Respiratory Pathogens from Bronchoalveolar Lavage Fluid Specimens.” Journal of Clinical Microbiology 60: e0052622. 10.1128/jcm.00526-22 35695488 PMC9297812

[11] Cabibbe, Andrea M. , Andrea Spitaleri , Simone Battaglia , Rebecca E. Colman , Anita Suresh , Anita Suresh , Swapna Uplekar , Timothy C. Rodwell , and Daniela M. Cirillo . 2020. “Application of Targeted Next‐Generation Sequencing Assay on a Portable Sequencing Platform for Culture‐Free Detection of Drug‐Resistant Tuberculosis from Clinical Samples.” Journal of Clinical Microbiology 58: 10. 10.1128/jcm.00632-20 PMC7512157 32727827

[12] Kambli, Priti , Kanchan Ajbani , Mubin Kazi , Meeta Sadani , Swapna Naik , Anjali Shetty , Jeffrey A. Tornheim , Harpreet Singh , and Camilla Rodrigues . 2021. “Targeted Next Generation Sequencing Directly from Sputum for Comprehensive Genetic Information on Drug Resistant *Mycobacterium tuberculosis* .” Tuberculosis 127: 102051. 10.1016/j.tube.2021.102051 33450448

[13] Quick, Joshua , Nathan D. Grubaugh , Steven T. Pullan , Ingra M. Claro , Andrew D. Smith , Karthik Gangavarapu , Glenn Oliveira , et al. 2017. “Multiplex PCR Method for MinION and Illumina Sequencing of Zika and Other Virus Genomes Directly From Clinical Samples.” Nature Protocols 12: 1261–1276. 10.1038/nprot.2017.066 28538739 PMC5902022

[14] Wu, Sheng‐Han , Yu‐Xin Xiao , Hseuh‐Chien Hsiao , and Ruwen Jou . 2022. “Development and Assessment of a Novel Whole‐Gene‐Based Targeted Next‐Generation Sequencing Assay for Detecting the Susceptibility of Mycobacterium Tuberculosis to 14 Drugs.” Microbiology Spectrum 10: e0260522. 10.1128/spectrum.02605-22 36255328 PMC9769975

[15] Cesar ignacio‐Espinoza, J , Sergei A. Solonenko , and Matthew B. Sullivan . 2013. “The Global Virome: Not as Big as we Thought?” Current Opinion in Virology 3: 566–571. 10.1016/j.coviro.2013.07.004 23896279

[16] Paez‐Espino, David , Emiley A. Eloe‐Fadrosh , Georgios A. Pavlopoulos , Alex D. Thomas , Marcel Huntemann , Natalia Mikhailova , Edward Rubin , Natalia N. Ivanova , and Nikos C. Kyrpides . 2016. “Uncovering Earth's Virome.” Nature 536: 425–430. 10.1038/nature19094 27533034

[17] Andreson, Reidar , Tõnu Möls , and Maido Remm . 2008. “Predicting Failure Rate of PCR In Large Genomes.” Nucleic Acids Research 36: e66. 10.1093/nar/gkn290 18492719 PMC2441781

[18] Bustin, Stephen A , Reinhold Mueller , and Tania Nolan . 2020. “Parameters for Successful PCR Primer Design.” Methods in Molecular Biology 2065: 5–22. 10.1007/978-1-4939-9833-3_2 31578684

[19] Leggate, Johanna , and Burton W Blais . 2006. “An Internal Amplification Control System Based on Primer‐Dimer Formation for PCR Product Detection By DNA Hybridization.” Journal of Food Protection 69: 2280–2284. 10.4315/0362-028x-69.9.2280 16995538

[20] Vallone, Peter M. , and John M. Butler . 2004. “AutoDimer: A Screening Tool for Primer‐Dimer and Hairpin Structures.” BioTechniques 37: 226–231. 10.2144/04372ST03 15335214

[21] Wang, Kun , Haiwei Li , Yue Xu , Qianzhi Shao , Jianming Yi , Ruichao Wang , Wanshi Cai , Xingyi Hang , Chenggang Zhang , Haoyang Cai et al. 2019. “MFEprimer‐3.0: Quality Control for PCR Primers.” Nucleic Acids Research 47: W610–W613. 10.1093/nar/gkz351 31066442 PMC6602485

[22] Ye, Jian , George Coulouris , Irena Zaretskaya , Ioana Cutcutache , Steve Rozen , and Thomas L Madden . 2012. “Primer‐BLAST: A Tool to Design Target‐Specific Primers for Polymerase Chain Reaction.” BMC Bioinformatics 13: 134. 10.1186/1471-2105-13-134 22708584 PMC3412702

[23] Guo, Jingwen , David Starr , and Huazhang Guo . 2021. “Classification and Review of Free PCR Primer Design Software.” Bioinformatics 36: 5263–5268. 10.1093/bioinformatics/btaa910 33104196

[24] Kechin, Andrey , Viktoria Borobova , Ulyana Boyarskikh , Evgeniy Khrapov , Sergey Subbotin , and Maxim Filipenko . 2020. “NGS‐PrimerPlex: High‐Throughput Primer Design for Multiplex Polymerase Chain Reactions.” PLoS Computational Biology 16: e1008468. 10.1371/journal.pcbi.1008468 33378360 PMC7802936

[25] Shen, Zhiyong , Wubin Qu , Wen Wang , Yiming Lu , Yonghong Wu , Zhifeng Li , Xingyi Hang , Xiaolei Wang , Dongsheng Zhao , and Chenggang Zhang . 2010. “MPprimer: A Program for Reliable Multiplex PCR Primer Design.” BMC Bioinformatics 11: 143. 10.1186/1471-2105-11-143 20298595 PMC2858037

[26] Untergasser, Andreas , Ioana Cutcutache , Triinu Koressaar , Jian Ye , Brant C Faircloth , Maido Remm , and Steven G Rozen . 2012. “Primer3‐‐New Capabilities and Interfaces.” Nucleic Acids Research 40: e115. 10.1093/nar/gks596 22730293 PMC3424584

[27] Bekaert, Michaël , and Emma C Teeling . 2008. “UniPrime: A Workflow‐Based Platform for Improved Universal Primer Design.” Nucleic Acids Research 36: e56. 10.1093/nar/gkn191 18424794 PMC2425486

[28] Collatz, Maximilian , Sascha D. Braun , Stefan Monecke , and Ralf Ehricht . 2022. “ConsensusPrime—A Bioinformatic Pipeline for Ideal Consensus Primer Design.” BioMedInformatics 2: 637–642. 10.3390/biomedinformatics2040041

[29] Fredslund, Jakob , Leif Schauser , Lene H. Madsen , Niels Sandal , and Jens Stougaard . 2005. “PriFi: Using a Multiple Alignment of Related Sequences to Find Primers for Amplification of Homologs.” Nucleic Acids Research 33: W516–W520. 10.1093/nar/gki425 15980525 PMC1160186

[30] Gadberry, Michael D. , Simon T. Malcomber , Andrew N. Doust , and Elizabeth A. Kellogg . 2005. “Primaclade—A Flexible Tool to Find Conserved PCR Primers Across Multiple Species.” Bioinformatics 21: 1263–1264. 10.1093/bioinformatics/bti134 15539448

[31] Hugerth, Luisa W. , Hugo A. Wefer , Sverker Lundin , Hedvig E. Jakobsson , Mathilda Lindberg , Sandra Rodin , Lars Engstrand , and Anders F. Andersson . 2014. “DegePrime, a Program for Degenerate Primer Design for Broad‐Taxonomic‐Range PCR in Microbial Ecology Studies.” Applied and Environmental Microbiology 80: 5116–5123. 10.1128/aem.01403-14 24928874 PMC4135748

[32] Jabado, Omar J. , Gustavo Palacios , Vishal Kapoor , Jeffrey Hui , Neil Renwick , Junhui Zhai , Thomas Briese , and W. Ian Lipkin . 2006. “Greene SCPrimer: A Rapid Comprehensive Tool for Designing Degenerate Primers from Multiple Sequence Alignments.” Nucleic Acids Research 34: 6605–6611. 10.1093/nar/gkl966 17135211 PMC1747188

[33] Kreer, Christoph , Matthias Döring , Nathalie Lehnen , Meryem S. Ercanoglu , Lutz Gieselmann , Domnica Luca , Kanika Jain , Philipp Schommers , Nico Pfeifer , and Florian Klein . 2020. “openPrimeR for Multiplex Amplification of Highly Diverse Templates.” Journal of Immunological Methods 480: 112752. 10.1016/j.jim.2020.112752 31991148

[34] Lamprecht, Anna‐Lena , Tiziana Margaria , Bernhard Steffen , Alexander Sczyrba , Sven Hartmeier , and Robert Giegerich . 2008. “GeneFisher‐P: Variations of GeneFisher as Processes in Bio‐jETI.” BMC Bioinformatics 9: S13. 10.1186/1471-2105-9-S4-S13 PMC2367627 18460174

[35] Lane, Courtney E. , Daniel Hulgan , Kelly O'Quinn , and Michael G. Benton . 2015. “CEMAsuite: Open Source Degenerate PCR Primer Design.” Bioinformatics 31: 3688–3690. 10.1093/bioinformatics/btv420 26198106

[36] Linhart, Chaim , and Ron Shamir . 2002. “The Degenerate Primer Design Problem.” Bioinformatics 18: S172–S181. 10.1093/bioinformatics/18.suppl_1.S172 12169545

[37] Rose, Timothy M. , Jorja G. Henikoff , and Steven Henikoff . 2003. “CODEHOP (COnsensus‐DEgenerate Hybrid Oligonucleotide Primer) PCR Primer Design.” Nucleic Acids Research 31: 3763–3766. 10.1093/nar/gkg524 12824413 PMC168931

[38] Yoon, Hyejin , and Thomas Leitner . 2015. “PrimerDesign‐M: A Multiple‐Alignment Based Multiple‐Primer Design Tool for Walking Across Variable Genomes.” Bioinformatics 31: 1472–1474. 10.1093/bioinformatics/btu832 25524896 PMC4410655

[39] You, Frank M. , Naxin Huo , Yong Q. Gu , Gerard R. Lazo , Jan Dvorak , and Olin D. Anderson . 2009. “ConservedPrimers 2.0: A High‐Throughput Pipeline for Comparative Genome Referenced Intron‐Flanking PCR Primer Design and Its Application in Wheat SNP Discovery.” BMC Bioinformatics 10: 331. 10.1186/1471-2105-10-331 19825183 PMC2765976

[40] Clarke, Erik L. , Sesh A. Sundararaman , Stephanie N. Seifert , Frederic D. Bushman , Beatrice H. Hahn , and Dustin Brisson . 2017. “Swga: A Primer Design Toolkit for Selective Whole Genome Amplification.” Bioinformatics 33: 2071–2077. 10.1093/bioinformatics/btx118 28334194 PMC5870857

[41] Gardner, Shea N. , Amy L. Hiddessen , Peter L. Williams , Christine Hara , Mark C. Wagner , and Bill W. Colston . 2009. “Multiplex Primer Prediction Software for Divergent Targets.” Nucleic Acids Research 37: 6291–6304. 10.1093/nar/gkp659 19759213 PMC2770652

[42] Hendling, Michaela , Stephan Pabinger , Konrad Peters , Noa Wolff , Rick Conzemius , and Ivan Barišić . 2018. “Oli2go: An Automated Multiplex Oligonucleotide Design Tool.” Nucleic Acids Research 46: W252–W256. 10.1093/nar/gky319 29718464 PMC6030895

[43] Wu, Yueni , Kai Feng , Ziyan Wei , Zhujun Wang , and Ye Deng . 2020. “ARDEP, a Rapid Degenerate Primer Design Pipeline Based on K‐Mers for Amplicon Microbiome Studies.” International Journal of Environmental Research and Public Health 17: 5958. 10.3390/ijerph17165958 32824566 PMC7459862

[44] Hysom, David A. , Pejman Naraghi‐Arani , Maher Elsheikh , A. Celena Carrillo , Peter L. Williams , and Shea N. Gardner . 2012. “Skip the Alignment: Degenerate, Multiplex Primer and Probe Design Using K‐Mer Matching Instead of Alignments.” PLoS One 7: e34560. 10.1371/journal.pone.0034560 22485178 PMC3317645

[45] Dwivedi‐Yu, Jane A. , Zachary J. Oppler , Matthew W. Mitchell , Yun S. Song , and Dustin Brisson . 2023. “A Fast Machine‐Learning‐Guided Primer Design Pipeline for Selective Whole Genome Amplification.” PLoS Computational Biology 19: e1010137. 10.1371/journal.pcbi.1010137 37068103 PMC10138271

[46] Huang, Yu‐Cheng , Chun‐Fan Chang , Chen‐Hsiung Chan , Tze‐Jung Yeh , Ya‐Chun Chang , Chaur‐Chin Chen , and Cheng‐Yan Kao . 2005. “Integrated Minimum‐Set Primers and Unique Probe Design Algorithms for Differential Detection on Symptom‐Related Pathogens.” Bioinformatics 21: 4330–4337. 10.1093/bioinformatics/bti730 16249263

[47] Wu, Jain‐Shing , Chungnan Lee , Chien‐Chang Wu , and Yow‐Ling Shiue . 2004. “Primer Design Using Genetic Algorithm.” Bioinformatics 20: 1710–1717. 10.1093/bioinformatics/bth147 14988099

[48] Wu, Jingli , Jianxin Wang , and Jian'er Chen . 2009. “A Practical Algorithm for Multiplex PCR Primer Set Selection.” International Journal of Bioinformatics Research and Applications 5: 38–49. 10.1504/ijbra.2009.022462 19136363

[49] Haas, Stefan , Martin Vingron , A. Poustka , and Stefan Wiemann . 1998. “Primer Design for Large Scale Sequencing.” Nucleic Acids Research 26: 3006–3012. 10.1093/nar/26.12.3006 9611248 PMC147651

[50] Riaz, Tiayyba , Wasim Shehzad , Alain Viari , François Pompanon , Pierre Taberlet , and Eric Coissac . 2011. “Ecoprimers: Inference of New DNA Barcode Markers from Whole Genome Sequence Analysis.” Nucleic Acids Research 39: e145. 10.1093/nar/gkr732 21930509 PMC3241669

[51] Smolander, Niina , Timothy R. Julian , and Manu Tamminen . 2022. “Prider: Multiplexed Primer Design Using Linearly Scaling Approximation of Set Coverage.” BMC Bioinformatics 23: 174. 10.1186/s12859-022-04710-1 35549665 PMC9097127

[52] Zhang, David Yu , Sherry Xi Chen , and Peng Yin . 2012. “Optimizing the Specificity of Nucleic Acid Hybridization.” Nature Chemistry 4: 208–214. 10.1038/nchem.1246 PMC4238961 22354435

[53] Banos, Stefanos , Guillaume Lentendu , Anna Kopf , Tesfaye Wubet , Frank Oliver Glöckner , and Marlis Reich . 2018. “A Comprehensive Fungi‐Specific 18S rRNA Gene Sequence Primer Toolkit Suited for Diverse Research Issues and Sequencing Platforms.” BMC Microbiology 18: 190. 10.1186/s12866-018-1331-4 30458701 PMC6247509

[54] Frech, Christian , Karin Breuer , Bernhard Ronacher , Thomas Kern , Christof Sohn , and Gerhard Gebauer . 2009. “Hybseek: Pathogen Primer Design Tool for Diagnostic Multi‐Analyte Assays.” Computer Methods and Programs in Biomedicine 94: 152–160. 10.1016/j.cmpb.2008.12.007 19201047

[55] Ghedira, Rim , Nina Papazova , Marnik Vuylsteke , Tom Ruttink , Isabel Taverniers , and Marc De Loose . 2009. “Assessment of Primer/Template Mismatch Effects on Real‐Time PCR Amplification of Target Taxa for GMO Quantification.” Journal of Agricultural and Food Chemistry 57: 9370–9377. 10.1021/jf901976a 19778057

[56] Rejali, Nick A. , Endi Moric , and Carl T. Wittwer . 2018. “The Effect of Single Mismatches on Primer Extension.” Clinical Chemisty 64: 801–809. 10.1373/clinchem.2017.282285 29444902

[57] Stadhouders, Ralph , Suzan D. Pas , Jeer Anber , Jolanda Voermans , Ted H. M. Mes , and Martin Schutten . 2010. “The Effect of Primer‐Template Mismatches on the Detection and Quantification of Nucleic Acids Using the 5′ Nuclease Assay.” The Journal of Molecular Diagnostics 12: 109–117. 10.2353/jmoldx.2010.090035 19948821 PMC2797725

[58] Zhao, Sheng , Cuicui Zhang , Liqun Wang , Minxuan Luo , Peng Zhang , Yue Wang , Waqar Afzal Malik , et al. 2023. “A Prolific and Robust Whole‐Genome Genotyping Method Using PCR Amplification Via Primer‐Template Mismatched Annealing.” Journal of Integrative Plant Biology 65: 633–645. 10.1111/jipb.13395 36269601

[59] Krausa, Peter , Julia G. Bodmer , and Michael J. Browning . 1993. “Defining the Common Subtypes of HLA A9, A10, A28 and A19 By Use of ARMS/PCR.” Tissue Antigens 42: 91–99. 10.1111/j.1399-0039.1993.tb02243.x 8266322

[60] Sint, Daniela , Lorna Raso , and Michael Traugott . 2012. “Advances in Multiplex PCR: Balancing Primer Efficiencies and Improving Detection Success.” Methods in Ecology and Evolution 3: 898–905. 10.1111/j.2041-210X.2012.00215.x 23549328 PMC3573865

[61] Linhart, Chaim , and Ron Shamir . 2005. “The Degenerate Primer Design Problem: Theory and Applications.” Journal of Computational Biology 12: 431–456. 10.1089/cmb.2005.12.431 15882141

[62] Mallona, Izaskun , Julia Weiss , and Marcos Egea‐Cortines . 2011. “PcrEfficiency: A Web Tool for PCR Amplification Efficiency Prediction.” BMC Bioinformatics 12: 404. 10.1186/1471-2105-12-404 22014212 PMC3234296

[63] Eisfeld, Amie J. , Gabriele Neumann , and Yoshihiro Kawaoka . 2014. “Influenza A Virus Isolation, Culture and Identification.” Nature Protocols 9: 2663–2681. 10.1038/nprot.2014.180 25321410 PMC5619698

[64] Köster, Johannes , and Sven Rahmann . 2012. “Snakemake‐‐A Scalable Bioinformatics Workflow Engine.” Bioinformatics 28: 2520–2522. 10.1093/bioinformatics/bts480 22908215

[65] Li, Weizhong , and Adam Godzik . 2006. “Cd‐Hit: A Fast Program for Clustering and Comparing Large Sets of Protein or Nucleotide Sequences.” Bioinformatics 22: 1658–1659. 10.1093/bioinformatics/btl158 16731699

[66] Hernández‐Salmerón, Julie E , and Gabriel Moreno‐Hagelsieb . 2022. “FastANI, Mash and Dashing Equally Differentiate Between Klebsiella Species.” PeerJ 10: e13784. 10.7717/peerj.13784 35891643 PMC9308963

[67] Edgar, Robert C . 2004. “MUSCLE: Multiple Sequence Alignment With High Accuracy and High Throughput.” Nucleic Acids Research 32: 1792–1797. 10.1093/nar/gkh340 15034147 PMC390337

[68] Katoh, Kazutaka , and Daron M. Standley . 2013. “MAFFT Multiple Sequence Alignment Software Version 7: Improvements in Performance and Usability.” Molecular Biology and Evolution 30: 772–780. 10.1093/molbev/mst010 23329690 PMC3603318

[69] Viterbi, A . 1967. “Error Bounds for Convolutional Codes and an Asymptotically Optimum Decoding Algorithm.” IEEE Transactions on Information Theory 13: 260–269. 10.1109/TIT.1967.1054010

[70] Kojima, Shigeyuki , Tsutomu Kageyama , Shuetsu Fukushi , Fuminori B. Hoshino , Michiyo Shinohara , Kazue Uchida , Katsuro Natori , Naokazu Takeda , and Kazuhiko Katayama . 2002. “Genogroup‐Specific PCR Primers for Detection of Norwalk‐Like Viruses.” Journal of Virological Methods 100: 107–114. 10.1016/s0166-0934(01)00404-9 11742657

[71] Nishida, Tomoko , Osamu Nishio , Masahiko Kato , Takehisa Chuma , Hirotomo Kato , Hiroyuki Iwata , and Hirokazu Kimura . 2007. “Genotyping and Quantitation of Noroviruses in Oysters from Two Distinct Sea Areas in Japan.” Microbiology and Immunology 51: 177–184. 10.1111/j.1348-0421.2007.tb03899.x 17310085

[72] Schultz, Anna Charlotte , Peter Saadbye , Jeffrey Hoorfar , and Birgit Nørrung . 2007. “Comparison of Methods for Detection of Norovirus in Oysters.” International Journal of Food Microbiology 114: 352–356. 10.1016/j.ijfoodmicro.2006.09.028 17182147

[73] Xie, Nina G. , Michael X. Wang , Ping Song , Shiqi Mao , Yifan Wang , Yuxia Yang , Junfeng Luo , Shengxiang Ren , and David Yu Zhang . 2022. “Designing Highly Multiplex PCR Primer Sets with Simulated Annealing Design Using Dimer Likelihood Estimation (SADDLE.” Nature Communications 13: 1881. 10.1038/s41467-022-29500-4 PMC9001684 35410464

[74] Langmead, Ben , and Steven L. Salzberg . 2012. “Fast Gapped‐Read Alignment with Bowtie 2.” Nature Methods 9: 357–359. 10.1038/nmeth.1923 22388286 PMC3322381

[75] Shen, Wei , Shuai Le , Yan Li , and Fuquan Hu . 2016. “SeqKit: A Cross‐Platform and Ultrafast Toolkit for FASTA/Q File Manipulation.” PLoS One 11: e0163962. 10.1371/journal.pone.0163962 27706213 PMC5051824

[76] Altschul, Stephen F. , Warren Gish , Webb Miller , Eugene W. Myers , and David J. Lipman . 1990. “Basic Local Alignment Search Tool.” Journal of Molecular Biology 215: 403–410. 10.1016/s0022-2836(05)80360-2 2231712

[77] Altschul, Stephen F. , Thomas L. Madden , Alejandro A. Schaffer , Jinghui Zhang , Zheng Zhang , Webb Miller , and David J. Lipman . 1997. “Gapped BLAST and PSI‐BLAST: A New Generation of Protein Database Search Programs.” Nucleic Acids Research 25: 3389–3402. 10.1093/nar/25.17.3389 9254694 PMC146917

[78] Li, Heng . 2018. “Minimap2: Pairwise Alignment for Nucleotide Sequences.” Bioinformatics 34: 3094–3100. 10.1093/bioinformatics/bty191 29750242 PMC6137996

