# Supplementary material for: MultiPrime: A reliable and efficient tool for targeted next‐generation sequencing
Source: Imeta. 2023 Oct 19;2(4):e143. doi: 10.1002/imt2.143 (PMC10989836; doi:10.1002/imt2.143)
Supplement: Supplementary file 1 — Supporting information. [file IMT2-2-e143-s002.docx]

**Supporting information to “MultiPrime A Reliable and Efficient Tool for Targeted Next-Generation Sequencing”**

Han Xia^1,2,3#^, Zhe Zhang^5#^, Chen Luo^3#^, Kangfei Wei^3#^, Xuming Li^3^, Xiyu Mu^3^, Meilin Duan^3^, Chuanlong Zhu^3^, Luyi Jin^3^, Xiaoqing He^3^, Lingjie Tang^3^, Long Hu^3^, Yuanlin Guan^3*^, David CC Lam^5*^, Junbo Yang^3,4,6*^

^1^School of Automation Science and Engineering, Faculty of Electronic and Information Engineering, Xi’an Jiaotong University, Xi’an, China

^2^MOE Key Lab for Intelligent Networks & Networks Security, Faculty of Electronic and Information Engineering, Xi’an Jiaotong University, Xi’an, China

^3^Department of Research and Development, Hugobiotech, Beijing, China.

^4^Shenzhen Branch, Guangdong Laboratory of Lingnan Modern Agriculture, Genome Analysis Laboratory of the Ministry of Agriculture and Rural Affairs, Agricultural Genomics Institute at Shenzhen, Chinese Academy of Agricultural Sciences, Shenzhen, Guangdong, China.

^5^Department of Mechanical and Aerospace Engineering, The Hong Kong University of Science and Technology, Clear Water Bay, Hong Kong, China

^6^Lead contact

^#^These authors contributed equally to this work: Han Xia, Zhe Zhang, Chen Luo, Kangfei Wei.

^*^Correspondence: 1806389316@pku.edu.cn (Junbo Yang); [medcclam@ust.hk](mailto:medcclam@ust.hk) (David CC Lam.); guanyuanlin@hugobiotech.com (Yuanlin Guan)

**
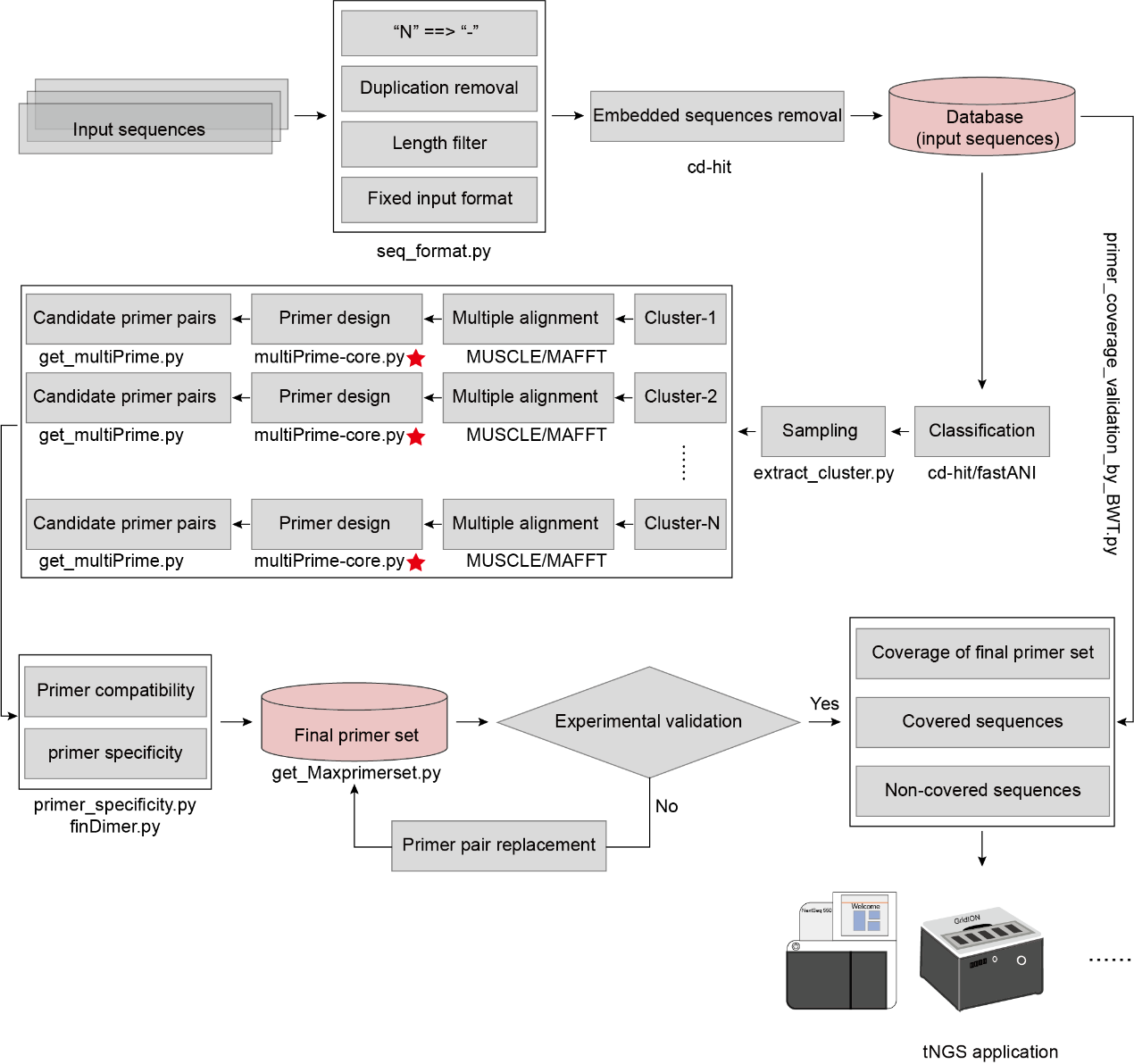
Supplemental Figures and Legends**

**Figure S1. Workflow of multiPrime.**

The red star indicates the step where the method is described in more detail.


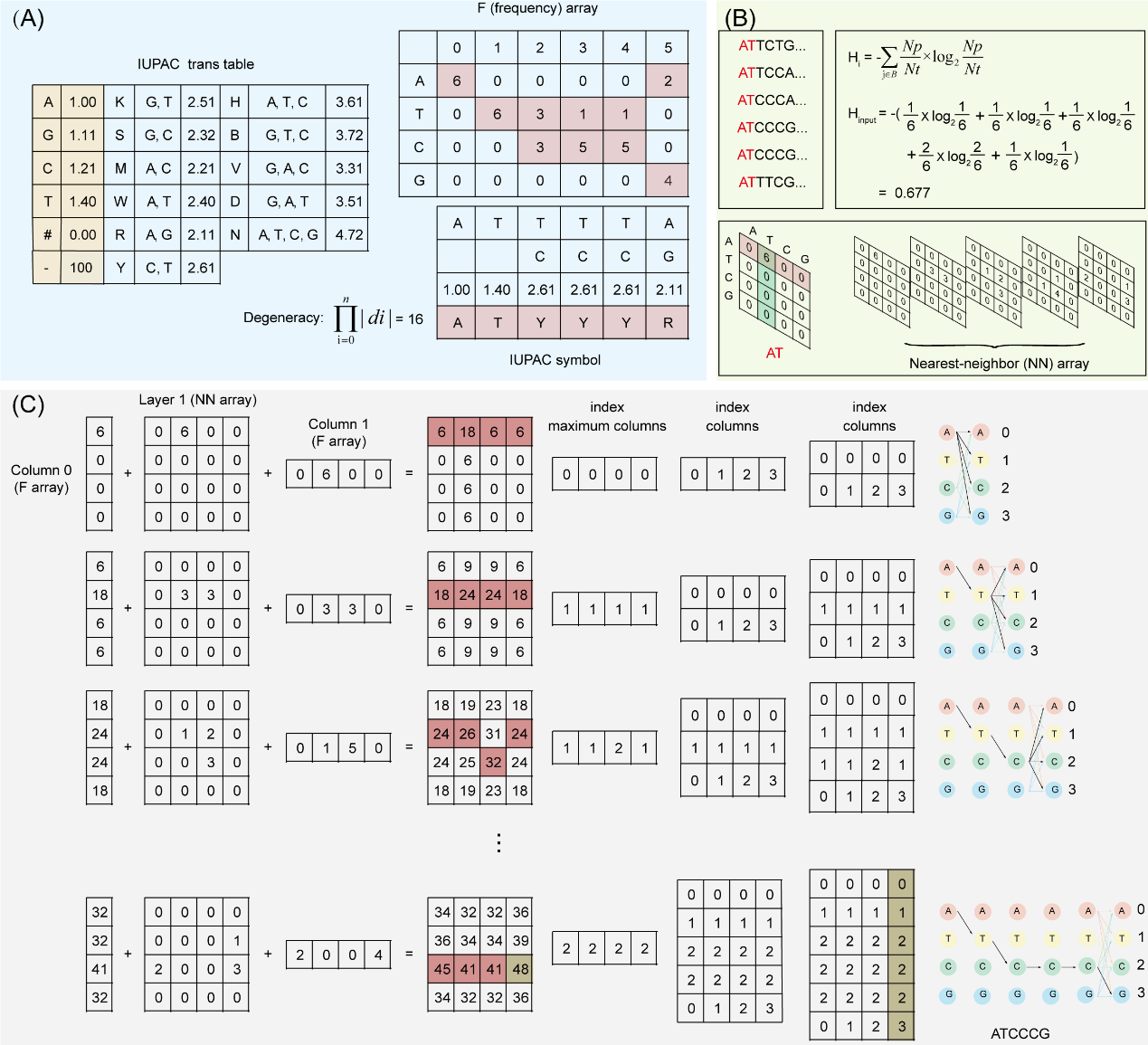
**Figure S2. IUPAC transition table and schematic of the Viterbi algorithm.**

A, Transition table and frequency array. The light-yellow highlighting indicates the normal base; the light-red highlighting indicates the frequency of nucleotides greater than 0 and the degenerate result through the IUPAC transition table. B, Calculation of entropy and the NN array. Hi: entropy, which represents the level of conservation of the window. C, Process of optimal primer selection with the Viterbi algorithm.

**
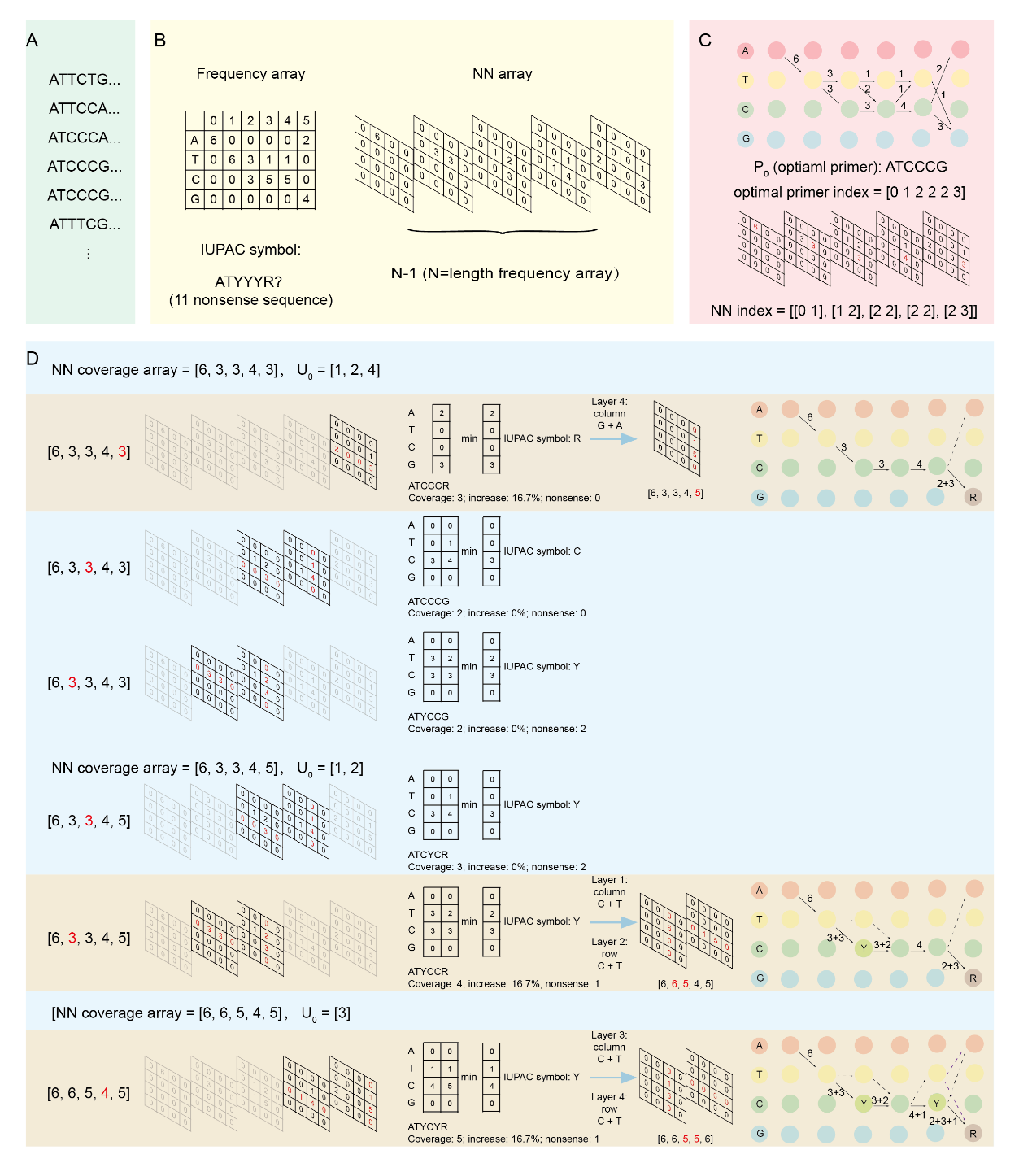
 Figure S3. Schematic of the NN model.** A, Input sequences. B, Frequency array and NN array preparation. The frequency array is the nucleotide frequency at each position of the window, while the NN array is the nearest-neighbor (NN) frequency of the window. “nonsense sequence” are primers that do not have a target sequence and are introduced through degenerate bases. C, The Viterbi algorithm is employed to find the most likely sequence of hidden states as the primary sequence to initialize degenerate primer design. D, A simplified process of the NN model. An NN array is created in the process of seeking a window (Supplementary Fig. 1B), and the row names and column names of the NN array are initially set to [A, T, C, G] by default. The NN index indicates the optimal primer coordinate. As shown in Fig. 2C, the optimal primer is ATCCCG, and the optimal primer coordinate is [0, 1, 2, 2, 2, 3]. Then, the NN index [[0, 1], [1, 2], [2, 2], [2, 2], [2, 3]] is obtained. The NN coverage array indicates the corresponding value of the NN index in the NN array ([6,3,3,4,3]). Let P0 be the optimal primer, with U0 representing the minimum set of positions in the NN coverage array. The minimum value in the NN coverage array is 3; then, U0 = [1, 2, 4], which denotes the 1st, 2nd and 4th layers of the NN array. There are three choices for the first round of optimization, and the best optimization site is selected. The minimum position in the NN coverage array is transformed to the IUPAC base; that is, the corresponding position of the NN array degenerates. For example, as shown in the first row of Fig. 2D, when U0 = 4 (the fourth layer of the NN index is [2, 3], which means that the second row and the third column of the fourth layer of the NN array need to degenerate), the second row is named C, and the third column is named G, corresponding to CG in the optimal primer (ATCC[CG]). The second row is [2,0,0,3], and the values in this row that are greater than 0 are 2 and 3; the corresponding indices are 0 and 3, which means that column 0 (column A) and column 3 (column G) are degenerate. In the IUPAC transition table, A corresponds to 1.00, G corresponds to 1.40, and the sum of the two normal bases is 2.40, which corresponds to R. Therefore, this position can be replaced by R. The optimal primer becomes ATCCCR, the primer coverage increases by 16.7%, and the number of nonsense primers is 0. Furthermore, the coverage of primers does not increase, and the number of nonsense primers increases by 2 when U0 = 1 or 2. Therefore, U0 = 4 is selected for optimization. The degenerate primer design with errors process is the same, except the coverage is replaced by coverage with errors. The NN array is refreshed after each iteration of refinement. For example, after column 0 is added to column 3, column 0 is subtracted from itself. Then, the column 3 name is replaced by the IUPAC symbol (column R), and column 0 becomes [0, 0, 0, 0].

**
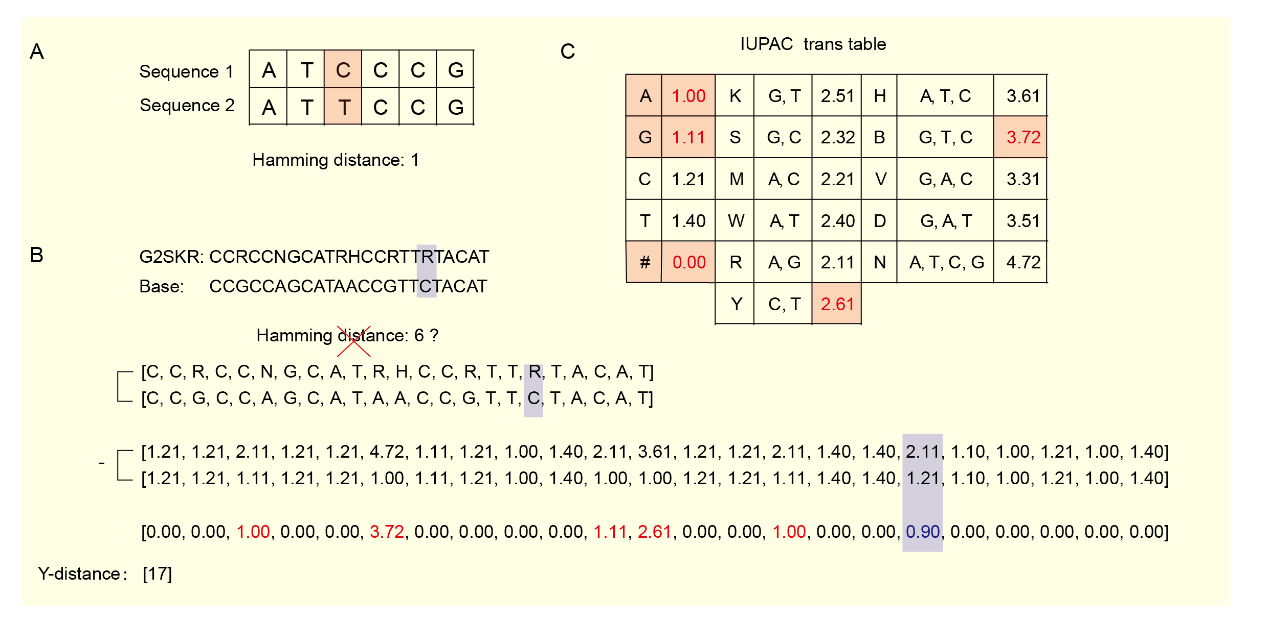
Figure S4. Evaluation of the distance between degenerate primer and target sequence.**

A, Hamming distance. The Hamming distance between two integers (strings) is the number of positions at which the corresponding bits are different. B, Y-distance. The Y-distance is an extension of the Hamming distance, and the Y-distance between two strings is the number of positions at the corresponding bits of the second string that are not contained in the first string. C, Calculation of the Y-distance. The position is recorded only when the difference value (array_G2SKR_ – array_Base_) is not present in Table C (blue in B); otherwise, the position will not be recorded (red in B and light red in C).

**
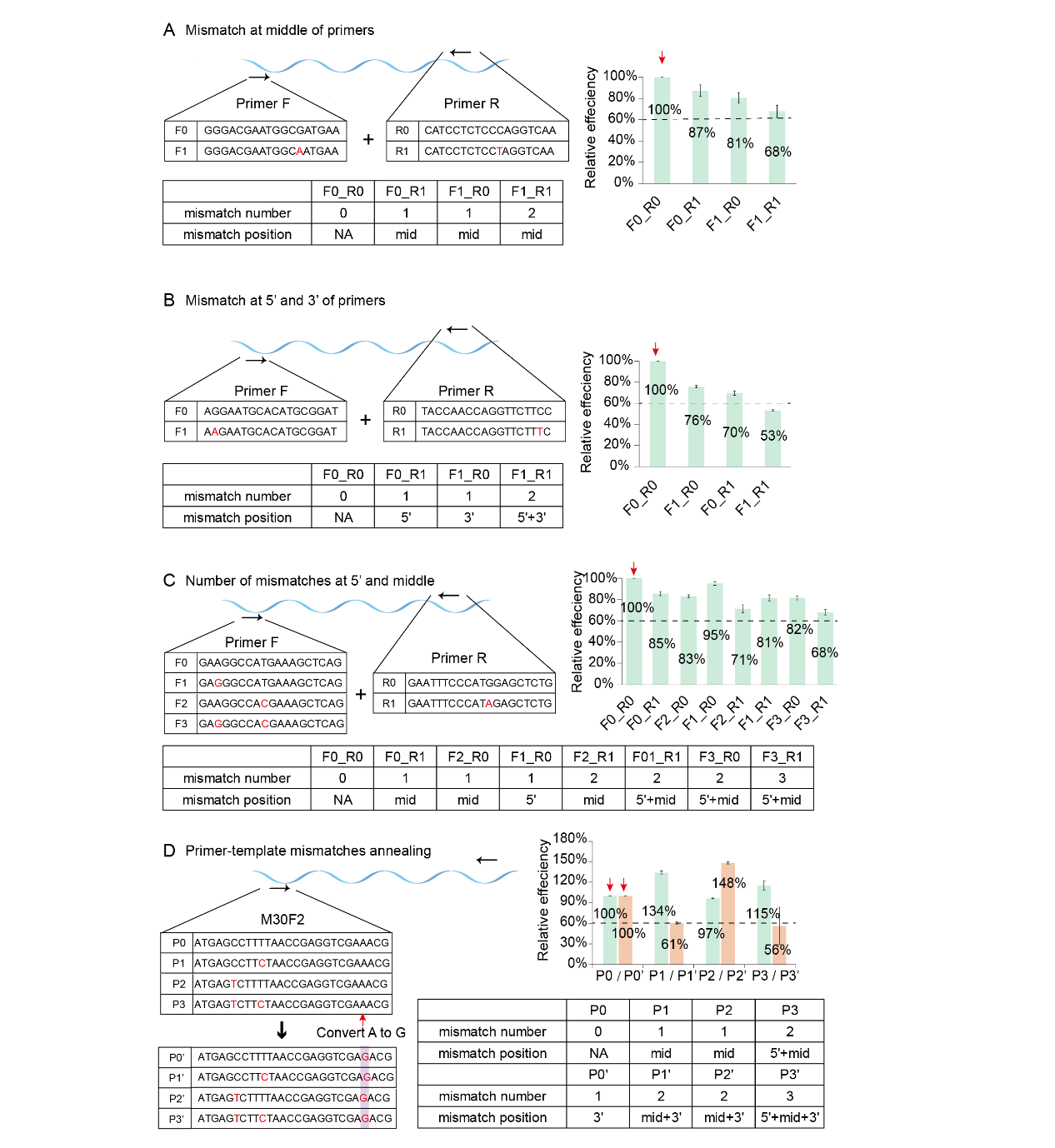
Figure S5**. **Successful application of target virus can still be achieved when primer-template mismatches annealing.** A, detection of influenza C with 0-1 mismatches in the middle region of primer. B, detection of influenza B with 0-1 mismatches in the 5’ and 3’ region of primer. C, detection of influenza B with 0-2 mismatches in the 5’ and middle positions of primer. D, detection of influenza A with 0-3 mismatches in the 5’, middle and 3’-adjacent regions of primer. Relative efficiency is defined as reads per million of primer divide by reads per million of primer-template perfect annealing primer. Red arrows in A-D indicate relative efficiency of primer-template perfect annealing primer. Data are represented as means ± SE (n = 3 biological replicates).

**
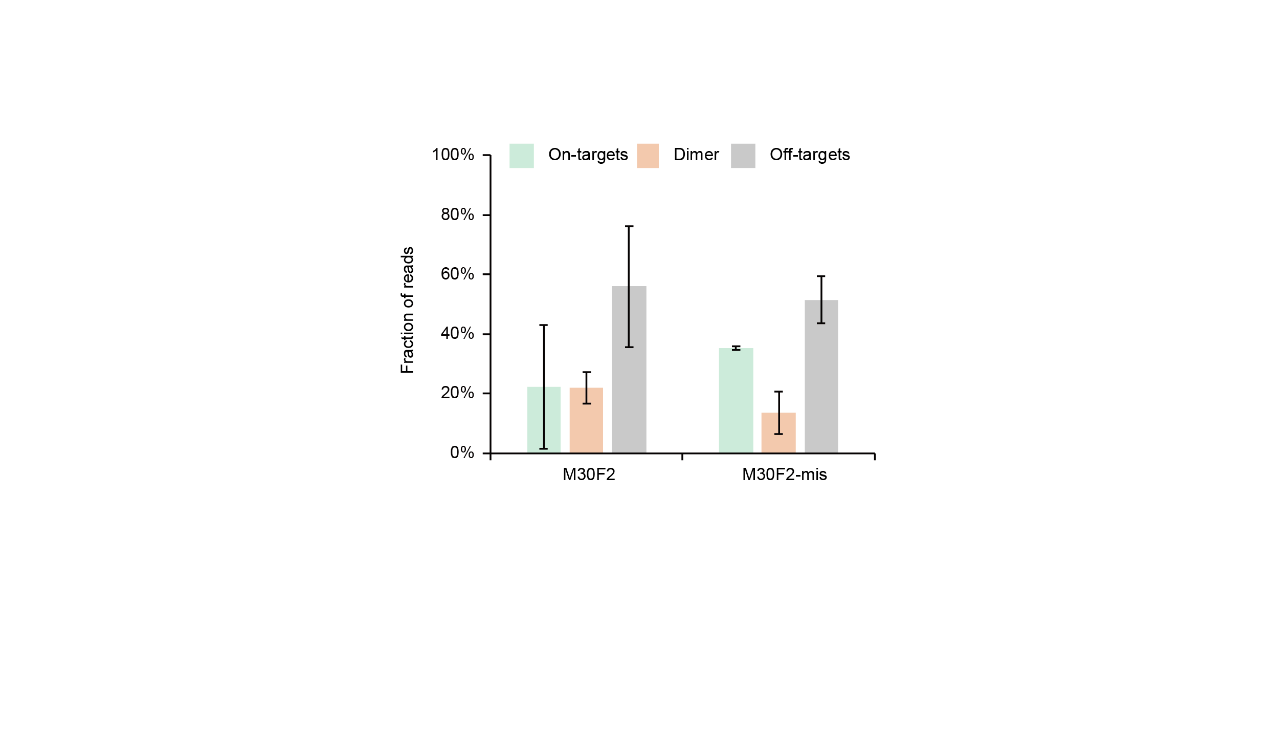
Figure S6. Distribution of reads observed in NGS library constructed using M30F2 and M30F2-mis.**

On-targets reads are defined as those that aligned to the target amplicons; Dimer are defined as dimer reads; all other reads were classified as Off-targets. Data are represented as means ± SE (n = 3 biological replicates).

**
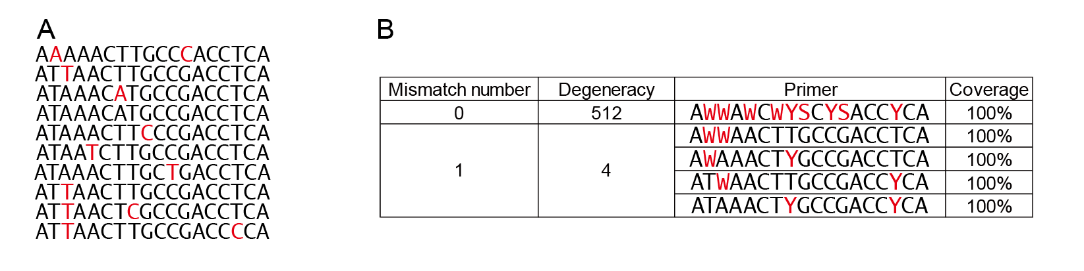
Figure S7. Degenerate primer with perfect match and allowing for 1 mismatch.**

Input sequences (A) and degenerate primers (B). The inclusion of 1 mismatch significantly decreases the primer degeneracy.


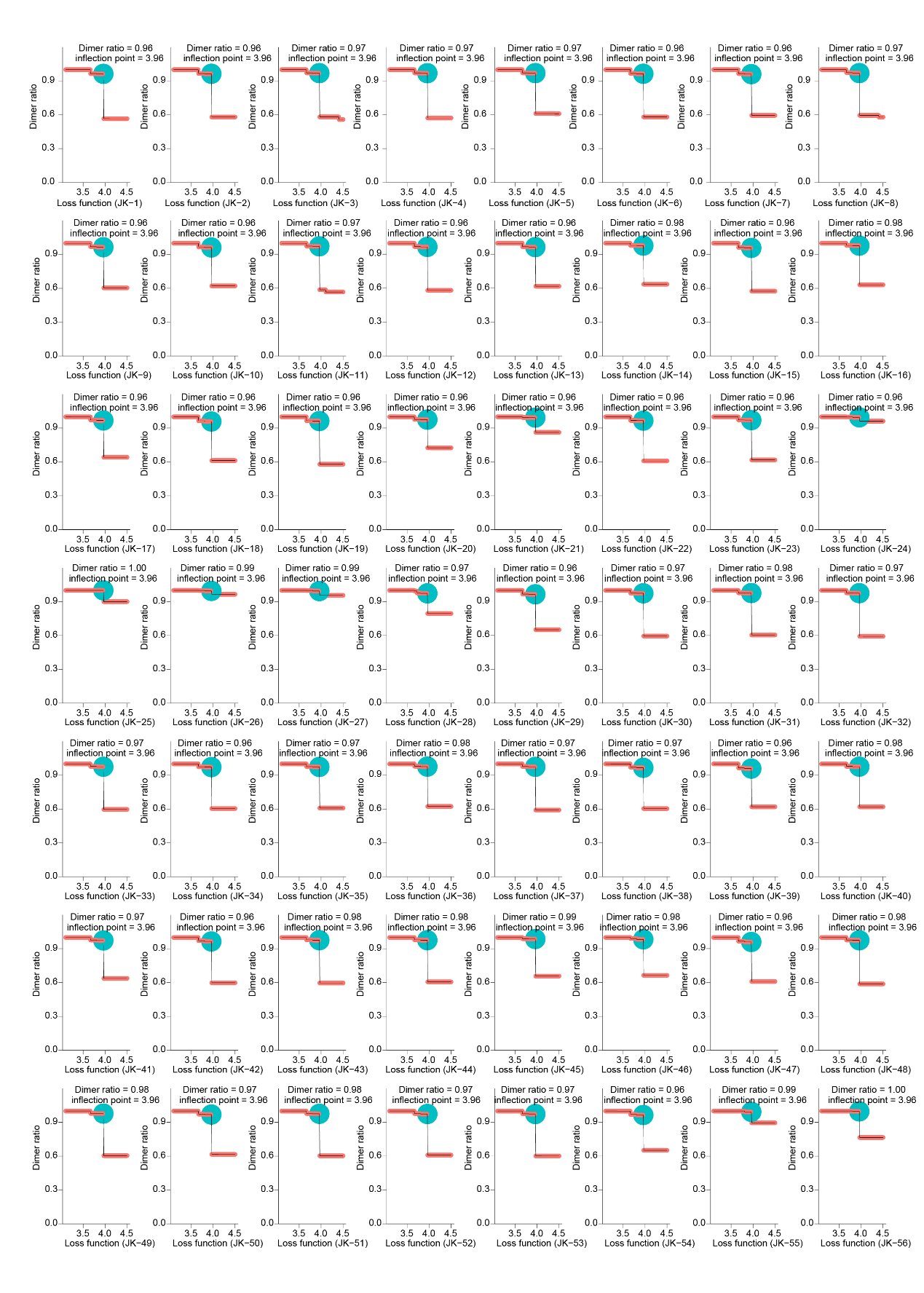


**
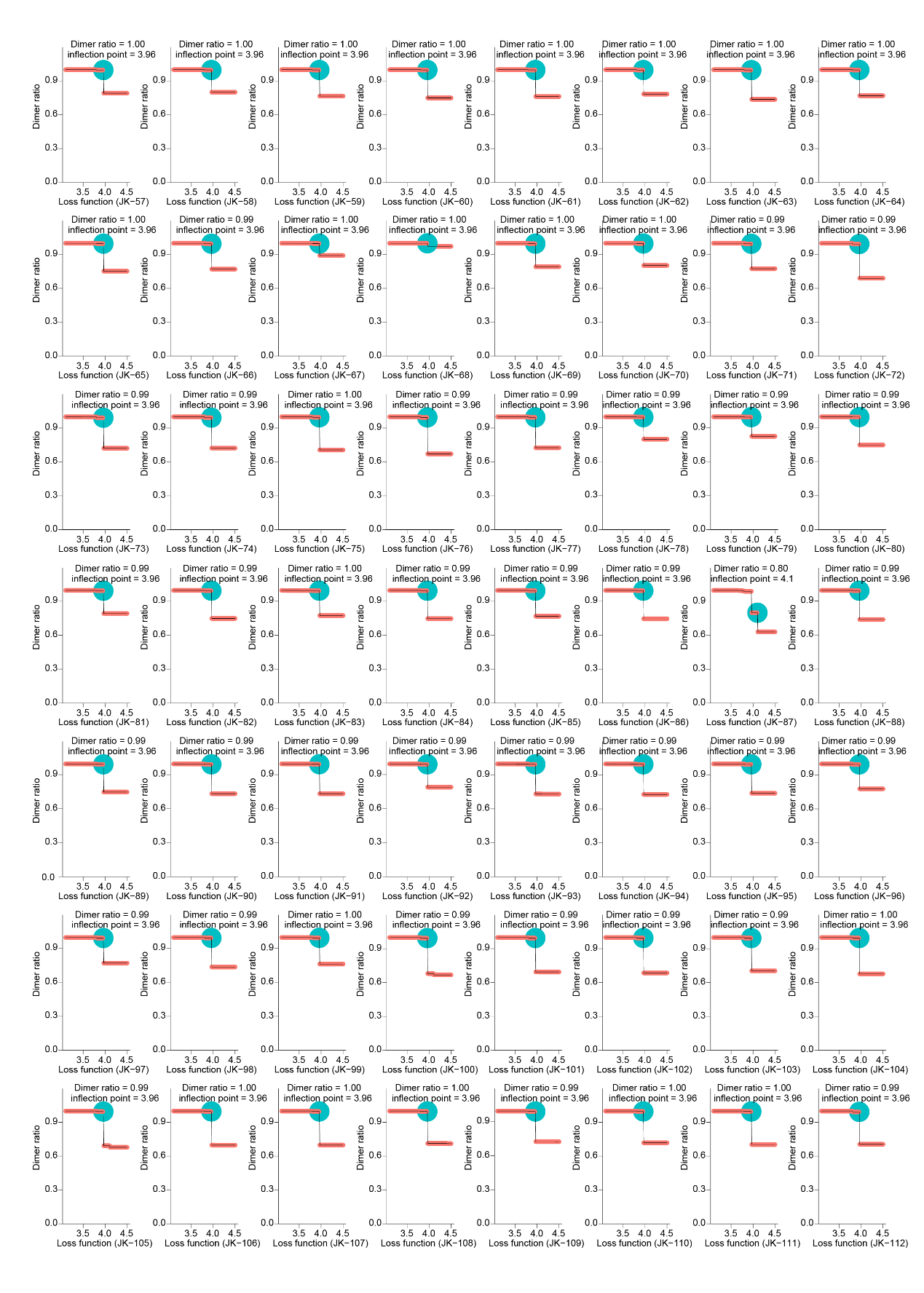
**

**
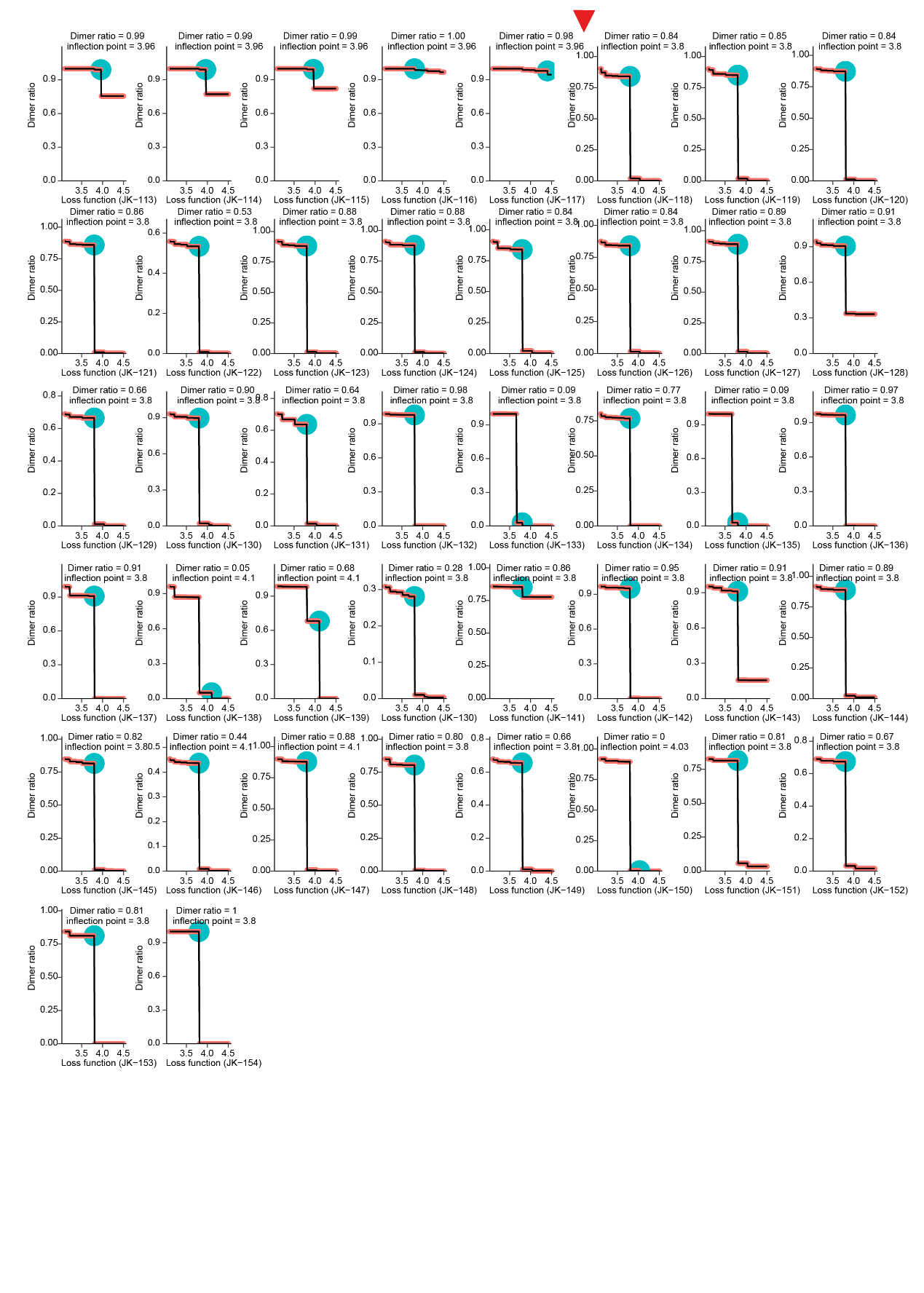
Figure S8.** **Dimer ratio of the 152 second-generation libraries.**

Dimer ratio refers to the percentage of reads number of primer pairs with a higher loss function value in the total dimer reads number of all the primer pairs (see methods). For example, the dimer ratio (loss function = 3.96) is the dimer number (those primer pairs with loss function > 3.96) divided by the total dimer number of all the primer pairs. Dimer examination was carried out on the libraries obtained from JK-117.

**
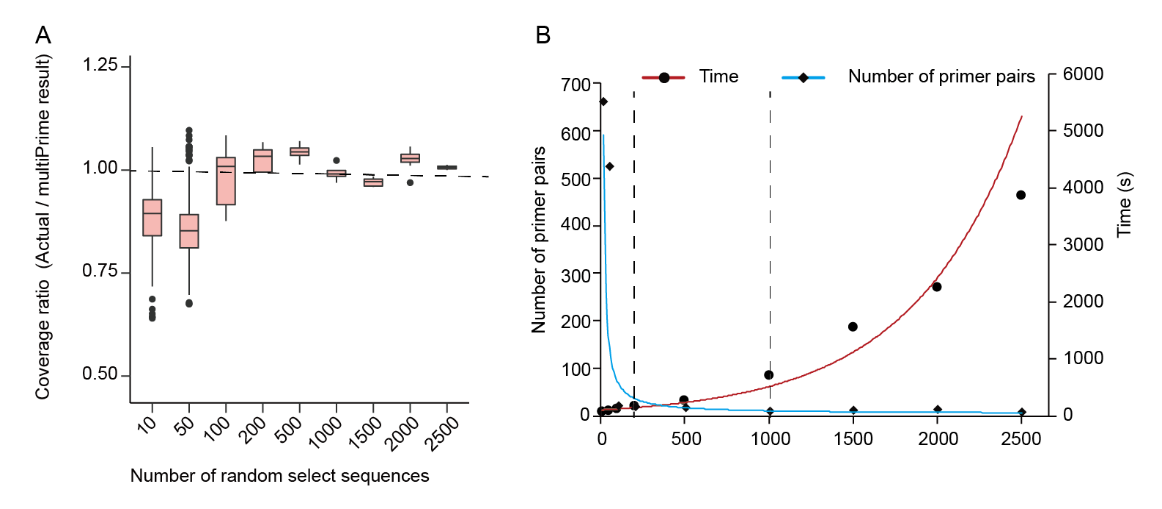
Figure S9. Optimization of multiPrime.**

A, the coverage ratio was calculated for random selections of sequences from 20732 CDSs of Influenza A. Coverage ratio indicates the actual coverage divided by the coverage achieved by the primer pairs designed by multiPrime. B, the number of primer pairs and time cost of multiPrime were evaluated using input sequences ranging from 10 to 2500.


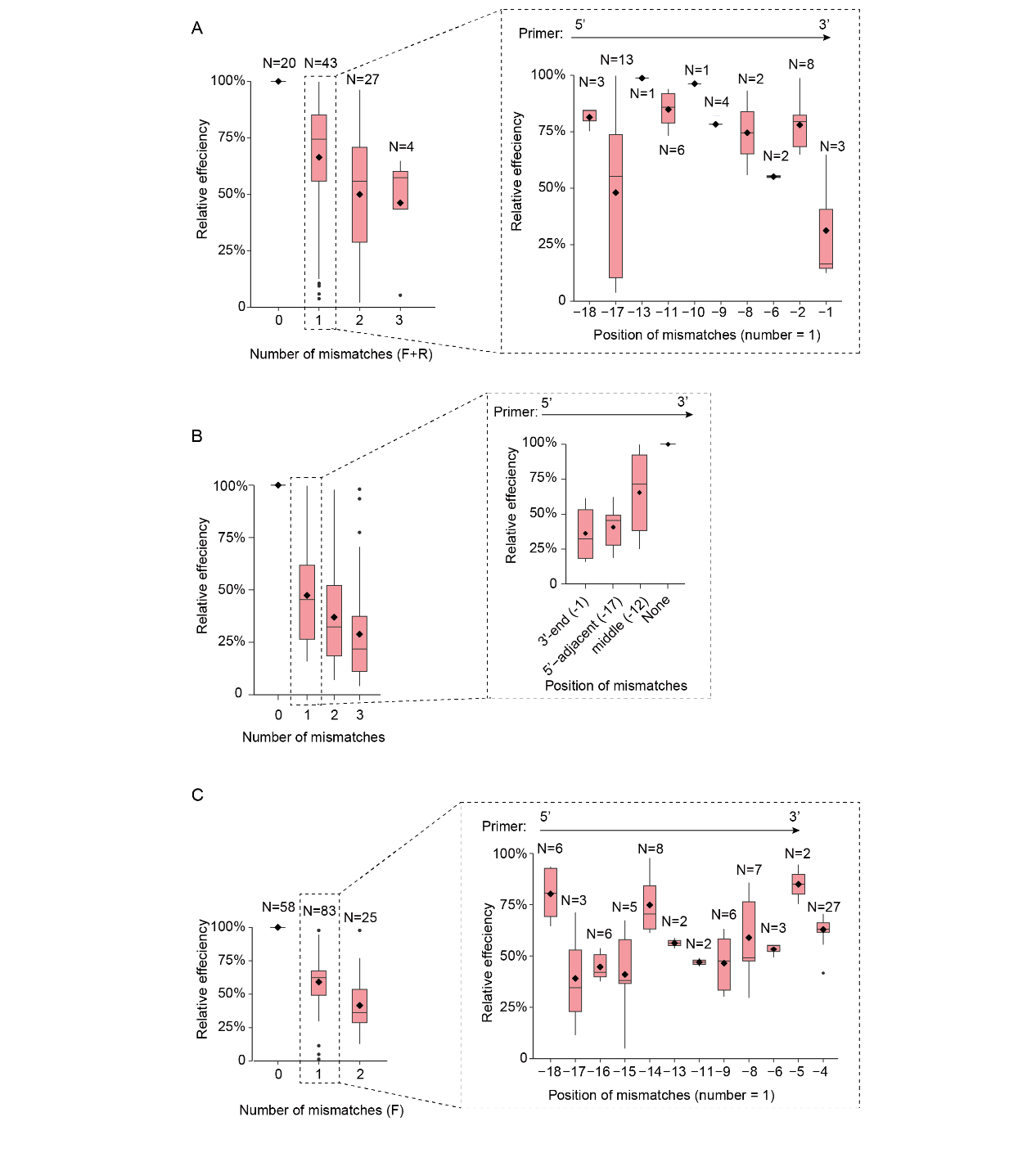
**Figure S10.** **Evaluation of primer efficiency**. A, relative efficiency of primers with 0-3 mismatch was evaluated on the ONT platform. F + R indicate indicate the total number of mismatches found in primer F and primer R, F indicate the total number of mismatches found in primer F. B, evaluation of primer effieiency with a synthesized DNA fragment (two biological replicates). C, relative efficiency of primers with 0-2 mismatch was evaluated on the NextSeq 500 platform.
